# Comparing methods for estimating patient‐specific treatment effects in individual patient data meta‐analysis

**DOI:** 10.1002/sim.8859

**Published:** 2020-12-27

**Authors:** Michael Seo, Ian R. White, Toshi A. Furukawa, Hissei Imai, Marco Valgimigli, Matthias Egger, Marcel Zwahlen, Orestis Efthimiou

**Affiliations:** ^1^ Institute of Social and Preventive Medicine University of Bern Bern Switzerland; ^2^ Graduate School for Health Sciences University of Bern Bern Switzerland; ^3^ MRC Clinical Trials Unit, Institute of Clinical Trials and Methodology University College London London UK; ^4^ Departments of Health Promotion and Human Behavior and of Clinical Epidemiology Kyoto University Graduate School of Medicine/School of Public Health Kyoto Japan; ^5^ Department of Cardiology, Bern University Hospital University of Bern Bern Switzerland

**Keywords:** Bayesian analysis, individual patient data, meta‐regression, shrinkage, variable selection

## Abstract

Meta‐analysis of individual patient data (IPD) is increasingly used to synthesize data from multiple trials. IPD meta‐analysis offers several advantages over meta‐analyzing aggregate data, including the capacity to individualize treatment recommendations. Trials usually collect information on many patient characteristics. Some of these covariates may strongly interact with treatment (and thus be associated with treatment effect modification) while others may have little effect. It is currently unclear whether a systematic approach to the selection of treatment‐covariate interactions in an IPD meta‐analysis can lead to better estimates of patient‐specific treatment effects. We aimed to answer this question by comparing in simulations the standard approach to IPD meta‐analysis (no variable selection, all treatment‐covariate interactions included in the model) with six alternative methods: stepwise regression, and five regression methods that perform shrinkage on treatment‐covariate interactions, that is, least absolute shrinkage and selection operator (LASSO), ridge, adaptive LASSO, Bayesian LASSO, and stochastic search variable selection. Exploring a range of scenarios, we found that shrinkage methods performed well for both continuous and dichotomous outcomes, for a variety of settings. In most scenarios, these methods gave lower mean squared error of the patient‐specific treatment effect as compared with the standard approach and stepwise regression. We illustrate the application of these methods in two datasets from cardiology and psychiatry. We recommend that future IPD meta‐analysis that aim to estimate patient‐specific treatment effects using multiple effect modifiers should use shrinkage methods, whereas stepwise regression should be avoided.

## 1 INTRODUCTION

Individual patient data (IPD) meta‐analysis of randomized clinical trials (RCTs) is often considered to be the best approach in evidence synthesis, despite being more resource‐intensive than the standard meta‐analysis based on aggregate data.^1^ IPD meta‐analysis is particularly valuable when there is interest in identifying which patients benefit most, since it achieves higher power than an aggregate meta‐analysis to detect differential treatment effects. Although standard meta‐analysis can be used to explore treatment‐covariate interaction (ie, by performing a “meta‐regression” on study‐level characteristics, eg, length of trial or mean age of participants), such analyses are hindered by the usually small number of studies in the analysis, by the typically small variation in study‐level characteristics and the threat of aggregation bias.^2^ By contrast, in an IPD meta‐analysis we model the individual outcome across many patients, with a usually much wider range in values of covariates. Furthermore, IPD meta‐analysis is less prone to aggregation bias as within trial information can be directly used to estimate how patient‐level characteristics modify treatment effects.^1^, ^3^, ^4^

There are two general approaches to perform IPD meta‐analysis: the one‐stage and the two‐stage approach. The two‐stage approach first analyzes each study separately, and then uses standard meta‐analysis methods to pool the effect of interest, such as the estimate of treatment effect and its SE. The one‐stage approach simultaneously models the individual participant data from all studies while respecting the randomization by accounting for the clustering of patients within each trial. One‐stage approaches may offer generally greater flexibility in modeling,^5^ although both approaches may lead to similar results in many cases.^6^

The usual goal of an IPD meta‐analysis is to estimate the (average) relative treatment effect, accounting for differences in the distribution of covariates among trials. Another goal is to identify possible treatment‐covariate interactions (ie, effect modification).^3^ If effect modification is present, there may be important clinical implications, that is, on whether to treat a particular patient subgroup. However, studies often collect large number of covariates, some of which might not interact with treatment, that is, when they do not modify treatment effect.^7^ Including all treatment‐covariate interactions in a model will often result in complex models, and may lead to overfitting and increased SEs.^8^ On the other hand, including too few interactions runs the risk of missing important characteristics that modify the patient‐specific treatment effect. Thus, methods for selecting which treatment‐covariate interactions to include in an IPD are of great interest.

Variable selection has a long history in statistics.^9^ Simple methods select variables based on a selection criterion such as the Akaike information criterion (AIC). For example, stepwise selection is popular, but controversial.^10^ More recent techniques such as least absolute shrinkage and selection operator (LASSO) have gained ground. LASSO shrinks coefficients and selects variables by forcing some of the coefficients to zero.^10^, ^11^, ^12^ Efficient algorithms have been developed to fit LASSO, such as the least angle regression.^11^ Similarly, methods allowing estimation of LASSO estimates for generalized linear models have become available.^12^

Bayesian LASSO imitates the frequentist LASSO, but has the advantage of readily providing SE estimates, which is not the case in classical LASSO unless approaches such as bootstrapping are used.^13^ Another penalization technique is ridge regression.^14^ One important difference is that ridge regression strictly does not select variables: it keeps all variables in the model and shrinks the corresponding coefficients.

Bayesian model averaging methods, such as stochastic search variable selection (SSVS) use a mixture prior on the regression coefficients and use indicator variables to select covariates in each step of the Markov Chain Monte Carlo (MCMC) iterations.^15^, ^16^ SSVS also does not actually select variables, but indicates how often the covariate has been included in the model.

It is unclear how these methods perform in IPD meta‐analysis, whether there is any benefit in using variable selection and/or shrinkage methods, and what is the optimal approach for IPD meta‐analysis. We hereby used simulations to compare different methods and formulate recommendations. We also illustrate the application of methods in two datasets from cardiology and psychiatry.

## 2 METHODS FOR VARIABLE SELECTION IN IPD META‐ANALYSIS

We start with simpler methods and then discuss approaches that are more advanced. We assume that studies have collected information on all covariates of interest, and that there are no missing outcomes or covariate data from all patients. For issues related to missing outcome data, we refer to the Discussion section.

### 2.1 Notation and general framework

We use *i* to denote a patient randomized in study *j* to receive treatment *t*_*i*_ (where *t*_*i*_ can be 0 or 1). For this patient we have information on a range of patient level covariates ***x***_***i***_. We use *m* to denote the different covariates included in ***x***_***i***_. We assume that before the analysis all continuous and binary covariates are centered around their means, so that ${\bar{x}}_{i}=0$. We denote the outcome of interest as *y*_*i*_ and focus on the case where *y*_*i*_ is either continuous or binary.

We can conceptually split the patient‐level covariates into three categories: covariates that have no effect on the outcome of interest (“nuisance covariates”), covariates that affect the outcome but do not interact with the treatment (“prognostic factors”), and covariates that affect the outcome and interact with treatment (“effect modifiers”). For example, if age is not related to *y* then age is a nuisance covariate. If age is related to *y* but does not have an interaction with treatment, then age is a prognostic factor. If age is related to *y* and interacts with the treatment, then age is an effect modifier. We denote the effect modifiers as $x_{i}^{\mathrm{EM}}$, where $x_{i}^{\mathrm{EM}}$ is a subset of ***x***_***i***_.

In what follows, we assume a linear relationship between the expected outcome (which may be transformed on some scale) and the patient covariates and interactions, and we will model this relationship using a generalized linear mixed effects model (GLMM). For the continuous case, we assume the outcome to be *y*_*ij*_ ∼ Normal(*μ*_*ij*_, *σ*^2^), where *μ*_*ij*_ (the “linear predictor”) denotes the expected outcome of the patient *i* randomized in study *j*, and *σ*^2^ refers to the variance of the outcome. The linear predictor is

(1) $$\mu_{\mathrm{ij}}=a_{j}+b_{j}x_{i}+{c_{j}x}_{i}^{\mathrm{EM}}t_{i}+d_{j}t_{i}.$$

In this equation *a*_*j*_ is a study‐specific intercept, vector ***b***_***j***_ includes the regression coefficients of the main effects of the covariates ***x***_***i***_, ***c***_***j***_ is the vector of coefficients for effect modifiers, and *d*_*j*_ is the treatment effect at ***x***_***i***_ = **0**, that is, at mean values of the covariates. For the treatment effect, covariates, and effect modifiers, we assume the following distributions:

(2) $$\begin{array}{l} d_{j}∼N\left(\delta ,\tau^{2}\right)\;\\ \;b_{j,m}∼N\left(\beta_{m},\tau_{\beta ,m}^{2}\right)\\ \;c_{j,m}∼N\left(\gamma_{m},\tau_{\gamma ,m}^{2}\right), \end{array}$$

where *δ* is the average underlying treatment effect, *τ*^2^ is the heterogeneity parameter of the treatment effect, *β*_*m*_ is the average main effect of covariate *m*,$\;\tau_{\beta ,m}^{2}$ is the heterogeneity parameter of the main effect of covariate *m*, *γ*_*m*_ is the average effect modification of covariate *m* and $\tau_{\gamma ,m}^{2}$ is the heterogeneity of the effect modification for covariate *m*. The study‐specific intercepts (*a*_*j*_) are assumed to be independent across studies.

Similarly, in the binary case, we assume the outcome to be generated from *y*_*ij*_ ∼ Bernoulli(*p*_*ij*_), where *p*_*ij*_ is probability of an event. The linear predictor on the log‐odds scale is

$$\log\left(\frac{p_{\mathrm{ij}}}{1-p_{\mathrm{ij}}}\right)=a_{j}+b_{j}x_{i}+{c_{j}x}_{i}^{\mathrm{EM}}t_{i}+d_{j}t_{i}$$

with the same considerations regarding the coefficients as noted above.

The usual goal of an IPD meta‐analysis is to estimate the average treatment effect *δ* in Equation (2), and to identify and quantify important treatment‐covariate interactions, that is, to estimate all *γ*_*m*_.

In what follows, we discuss methods that perform variable selection and/or shrinkage in IPD meta‐analysis. In all methods we discuss selection and/or shrinkage is only applied to covariate‐by‐treatment interaction terms, whereas the main effects of the covariates are always included in the models without any shrinkage.

### 2.2 Generalized linear mixed effects model (GLMM‐full and GLMM‐oracle)

The simplest approach for analyzing such data is via a GLMM with all covariates and treatment‐covariate interactions included (“GLMM‐full”), that is, to fit the model where the linear predictor includes all available treatment‐covariate interactions. Note that in practice, estimating all the different heterogeneity parameters included in Equation (2) might be hard, especially when there are only few studies available, or when (for the case of a binary outcome) the event rate is low. For this reason, we might often assume common regression coefficients for the main effect and effect modification, that is, *b*_*j*, *m*_ = *β*_*m*_ and *c*_*j*, *m*_ = *γ*_*m*_ for all studies.

In our simulations (Section 3) we will also use the “GLMM‐oracle,” that is, a GLMM that includes only the main effects and treatment‐covariate interactions that were used to generate the simulated data.

### 2.3 Stepwise variable selection (STEP)

Stepwise variable selection has been widely used in the past. There are three different flavors of stepwise selection, depending on the directionality of the selection procedure: “forward,” “backward,” and “bidirectional.” Forward stepwise regression starts from a base model (ie, with no effect modifiers), considers all one‐variable expansions of the model, and adds the effect modifiers according to some prespecified criterion. Possible criteria include the AIC, the Bayesian information criterion (BIC), or the *P*‐value of the interaction terms. The process continues until the value of the criterion stops improving. Backward stepwise regression starts from the full model and eliminates effect modifiers according to a criterion. Bidirectional stepwise regression considers both adding and removing one variable at each step, and takes the best option according to the criterion.^9^, ^17^ Stepwise regression has been criticized for bias in estimating the coefficients because of the so‐called “testimation bias” or “winner's curse.”^18^

### 2.4 Least absolute shrinkage and selection operator

LASSO regression aims to reduce model complexity and prevent overfitting.^10^ The model uses a *L*_1_ penalty term in the optimization function, controlled by a penalty parameter *λ*. The inclusion of the penalty term leads to a shrinkage of the effect modification. Some of the coefficients may shrink to zero leading to the exclusion of the corresponding interaction terms from the model. Thus, different values of *λ* correspond to different models. All effect modifiers are penalized equally, so all patient characteristics need to be standardized a priori (ie, by dividing with the corresponding SD). For a continuous outcome, the objective (given *λ*) is to identify the values of the coefficients which minimize the following quantity

(3) $$\min_{a_{j},\beta ,\gamma ,\delta }\frac{1}{2N}\sum_{i=1}^{N}{\left(y_{\mathrm{ij}}-\left(a_{j}+\beta x_{i}+\gamma x_{i}t_{i}+\delta t_{i}\right)\right)}^{2}+\lambda \left[\sum_{m=1}^{p},|,\gamma_{m},|,\right],$$

where *a*_*j*_ is the study‐specific intercept, ***β*** is the average main effect of covariate, ***γ*** is the average effect modification of covariate, *γ*_*m*_ is the average effect modification of covariate *m*, *N* is total number of patients, and *p* is total number of covariates. Similarly, for a dichotomous outcome, the objective is to minimize

$$\min_{a_{j},\beta ,\gamma ,\delta }-\left[\frac{1}{N}\sum_{i=1}^{N}y_{\mathrm{ij}}\times \left(a_{j}+\beta x_{i}+\gamma x_{i}t_{i}+\delta t_{i}\right)-\log\left(1+e^{a_{j}+\beta x_{i}+\gamma x_{i}t_{i}+\delta t_{i}}\right)\right]+\lambda \left[\sum_{m=1}^{p},|,\gamma_{m},|,\right].$$

The value of *λ* can be determined by *k*‐fold cross‐validation, but can also be chosen by minimizing AIC or BIC.^19^ *k*‐fold cross‐validation randomly divides the sample into *k* subsamples with equal number of observations. Out of the *k* subsamples, one subsample is used as a testing set and the others are used as a training set, to calculate a cross‐validation error. The procedure continues by cycling through all *k* subsamples. A common choice for *k* is 5 or 10, but other values can be used. For a discussion on the choice of *k* we refer our readers to section 7.10 of the book by Hastie et al.^19^ The choice of *k* may be decided considering sample size. This cross‐validation is repeated for multiple values of *λ*. The value that minimizes the cross‐validation error is selected for the final model. For a Gaussian outcome, deviance (ie, squared error) can be used for the cross‐validation error. For a binary outcome, the deviance from logistic regression, the misclassification rate or the area under the curve can be used to assess the cross‐validation error. Note that for the case of area under the curve, we would be maximizing (rather than minimizing) the metric, in order to choose *λ*.

Also note that in Equation (3), we use the average treatment effect *δ*, instead of *d*_*j*_ (the study‐specific treatment effect in Equation (2)). Likewise for ***β***, ***γ***. Thus, this model assumes common treatment effect, main effect, and effect modification, that is, it does not account for between‐study heterogeneity. Regarding a random‐effects version of LASSO we refer our readers to the discussion section.

No simple formula for SEs exists for LASSO. The bootstrap method^20^ can instead be used to estimate SEs.^10^ However, this approach has been criticized for failing to be consistent, and alternative methods such as residual bootstrap methods have been proposed.^21^, ^22^ Furthermore, there is an ongoing research on postselection inference for LASSO, aiming to form valid confidence intervals for the selected coefficients after model selection.^23^, ^24^

### 2.5 Ridge regression (ridge)

Ridge regression^14^ is in many ways similar to LASSO. Despite its inability to produce a parsimonious model (because it keeps all treatment‐covariate interactions in the model), ridge regression has been shown to lead to better prediction models than LASSO in some situations, for example, when predictors are highly correlated.^25^ Ridge regression uses a *L*_2_ penalty term (ie, squared magnitude) in the optimization function. For a continuous outcome, the objective is (given *λ*) to minimize

$$\min_{a_{j},\beta ,\gamma ,\delta }\frac{1}{2N}\sum_{i=1}^{N}{\left(y_{\mathrm{ij}}-\left(a_{j}+\beta x_{i}+\gamma x_{i}t_{i}+\delta t_{i}\right)\right)}^{2}+\lambda \left[\sum_{m=1}^{p}\gamma_{m}^{2}\right].$$

Similarly, for dichotomous outcome, the objective is to minimize

$$\min_{a_{j},\beta ,\gamma ,\delta }-\left[\frac{1}{N}\sum_{i=1}^{N}y_{\mathrm{ij}}\times \left(a_{j}+\beta x_{i}+\gamma x_{i}t_{i}+\delta t_{i}\right)-\log\left(1+e^{a_{j}+\beta x_{i}+\gamma x_{i}t_{i}+\delta t_{i}}\right)\right]+\lambda \left[\sum_{m=1}^{p}\gamma_{m}^{2}\right].$$

The selection of the *λ* follows the same considerations as in LASSO. One distinct feature of ridge regression is that, contrary to LASSO, for ridge regression, there is a closed form solution for the estimates and the SE, when the outcome is normally distributed, given *λ*.^26^ Note that, as was the case for LASSO, this model cannot readily include random effects structure.

### 2.6 Adaptive LASSO

Adaptive LASSO^27^ is a modified version of LASSO, where adaptive weights are used for penalizing different coefficients in the *L*_1_ penalty. Estimates of adaptive LASSO are given by

$$\min_{a_{j},\beta ,\gamma ,\delta }\frac{1}{2N}\sum_{i=1}^{N}{\left(y_{\mathrm{ij}}-\left(a_{j}+\beta x_{i}+\gamma x_{i}t_{i}+\delta t_{i}\right)\right)}^{2}+\lambda \left[\sum_{m=1}^{p}{\hat{w}}_{m},|,\gamma_{m},|,\right].$$

Similarly, for dichotomous outcome, estimates are given by

$$\min_{a_{j},\beta ,\gamma ,\delta }-\left[\frac{1}{N}\sum_{i=1}^{N}y_{\mathrm{ij}}\times \left(a_{j}+\beta x_{i}+\gamma x_{i}t_{i}+\delta t_{i}\right)-\log\left(1+e^{a_{j}+\beta x_{i}+\gamma x_{i}t_{i}+\delta t_{i}}\right)\right]+\lambda \left[\sum_{m=1}^{p}{\hat{w}}_{m},|,\gamma_{m},|,\right],$$

where all parameters follow the same definition from above sections and ${\hat{w}}_{m}$ is a weight vector defined as ${\hat{w}}_{m}=1/{\hat{\gamma }}_{m}^{l}$. In this formula, ${\hat{\gamma }}_{m}$ can be any root‐*n* consistent estimator such as LASSO, ridge, or least squares estimate. *l* is often set equal to 1, but could also be estimated using cross‐validation.

### 2.7 Bayesian LASSO with mixed effects (Bayesian LASSO)

Regarding the optimization problem in Equation (3), Tibshirani^10^ noted that LASSO estimate can be viewed as the mode of the posterior distribution in a Bayesian analysis, when the regression coefficients are assigned a Laplace (ie, double exponential) prior. Park and Casella^13^ pursued the idea further and introduced the Bayesian LASSO that uses the conditional Laplace prior

(4) $$\pi \left(\gamma |\sigma \right)=\prod_{m=1}^{p}\frac{\lambda }{2\sigma }e^{-\lambda |\gamma_{m}|/\sigma },$$

where *p* is the number of covariates included in ***x***. In this formula, *σ* is the SD of the continuous outcome. For a binary outcome, we use an unconditional Laplace prior given as

$$\pi \left(\gamma \right)=\prod_{m=1}^{p}\frac{\lambda }{2}e^{-\lambda |\gamma_{m}|}.$$

Using these prior distributions, we obtain shrunk estimates of the effect modifiers in a logistic regression.^28^ A Laplace prior has a peak around zero and, compared with a Gaussian prior, allows small coefficients to shrink towards zero faster, while it applies smaller shrinkage to large coefficients.

Note that the Bayesian LASSO model can allow for a random effect structure on all, or some of the coefficients. However, it might be difficult to estimate heterogeneity precisely when there are a few studies^29^ or when there is a rare binary outcome. In such cases the posterior of *τ* will be heavily influenced by the prior, for example, leading to excessively large SEs for the case of “vague” priors. Also of note, there are several ways to select the shrinkage parameter *λ*. We can use cross‐validation, treat it as random and assign a noninformative hyperprior,^13^ or choose the value via Bayes factors.^30^ An advantage of using Bayesian LASSO is that standard errors are readily available. One limitation is that Bayesian LASSO does not strictly perform variable selection, since covariates are not shrunk all the way to zero.

### 2.8 Stochastic search variable selection

First proposed by George and McCulloch,^31^ this Bayesian model introduces indicator variables that allow the selection of covariates at each step of the MCMC iterations. In this model, we use a mixture prior on *γ*_*m*_, the average effect modification of covariate *m*

$$P\left(\gamma_{m}|I_{m}\right)=\left(1-I_{m}\right)N\left(0,\eta^{2}\right)+I_{m}N\left(0,g\eta^{2}\right),$$

where the variable *I*_*m*_ can take values 0 or 1 and is used to indicate the absence or presence of the interaction term of covariate *m* in the model. *η*^2^ is the variance when the interaction term is absent, and *g* is a factor multiplied to *η*^2^ for the case when the interaction term is present. Thus, if *η*^2^ is assigned a small value, the prior for the effect modifier is a narrow distribution concentrated around 0, for the case when *I*_*m*_ = 0, that is, when the interaction term is absent. Assigning a large value for *g* allows a flat prior for the interaction term when *I*_*m*_ = 1, that is, when the covariate is included in the model as an effect modifier. We can assign *I*_*m*_ to have a Bernoulli prior with probability .5, which makes all models equally probable.^31^ Alternatively, expert opinion can be used to formulate informative priors for each *I*_*m*_. Meuwissen and Goddard^32^ introduced a variant of SSVS where *η*^2^ was assumed random and estimated in the model with a prior and *g* fixed at 100.^32^ Similarly to the Bayesian LASSO, strictly speaking there is no variable selection performed. Rather, model averaging is performed, as determined by the posterior distribution of *I*_*m*_. The mean values of the posterior of the indicators tell us how often each effect modifier has been included in the model.

## 3 SETUP FOR THE SIMULATION STUDY

In this section, we describe a simulation study aimed to compare the methods for estimating patient‐specific treatment effects in IPD meta‐analysis outlined in the previous section. We describe the various scenarios explored, the approaches compared, and the methods used to assess their performance.

### 3.1 Data generating mechanism

We examined continuous and binary outcomes and used Equations (1) and (2) to simulate data. In order to generate the data, we followed six steps:

- For each study, we generated the total number of patients by drawing from Uniform(50,100). Then, we generated the patient‐level covariates ***x***_***i***_ for each patient *i*. For continuous covariates we sampled from *N*_*p*_(0, ***∑***), where *p* was the total number of covariates, and the (*i*, *j*) element of ***∑*** was equal to *ρ*^∣*i* − *j*∣^. The correlation coefficient parameter (*ρ*) was set to 0.3. For binary covariates, we sampled from Bernoulli(0.5). The number of studies and the number of covariates included in ***x***_***i***_ (and their type, ie, nuisance covariates vs prognostic factors vs effect modifiers) depended on the scenario (see following section).
- We generated the treatment indicator *t*_*i*_ by sampling from a Bernoulli(0.5) as we focused on simulating data from randomized trials.
- We generated the study‐specific treatment effect of each study *d*_*j*_ by drawing from *N*(*δ*, *τ*^2^) where the average treatment effect *δ* was set to be equal to 1 for all scenarios, and *τ* depended on the scenario.
- We generated the study‐specific main effect of covariate *m*, *b*_*j*, *m*_, by drawing from $N\left(\beta_{m}\tau_{\beta ,m}^{2}\right)$; likewise, we generated the study‐specific effect modification *c*_*j*, *m*_ by drawing from $N\left(\gamma_{m}\tau_{\gamma ,m}^{2}\right)$. We set *τ*_*β*, *m*_ = 0.2, *τ*_*γ*, *m*_ = 0.3, and *β*_*m*_ = 0.2. The values of *γ*_*m*_ depended on the scenario.
- We generated independent study‐specific intercepts (*α*_*j*_) by sampling from Uniform(−1, 1) for the continuous outcome and from Uniform(−2, −1) for a binary outcome. The latter was chosen to obtain datasets with event rates around 30%. This was needed to ensure smooth fitting of all models; an initial exploratory analysis showed that very small (or very large) event rates led to estimation problems, for example, when some of the simulated datasets had no events in one of the treatment arms for specific levels of the covariates.
- Finally, we generated *y*_*i*_ by drawing from$\;\text{Bernoulli}\left(\text{logi}t^{-1}\left(a_{j}+b_{j}x_{i}+{c_{j}x}_{i}^{\mathrm{EM}}t_{i}+d_{j}t_{i}\right)\right)$ for the binary outcome and $\text{Normal}\left(a_{j}+b_{j}x_{i}+{c_{j}x}_{i}^{\mathrm{EM}}t_{i}+d_{j}t_{i}\sigma =0.5\right))$ for the continuous outcome.

### 3.2 Simulations scenarios and models compared

We explored 72 different scenarios. We used both binary and continuous outcomes and we explored different configurations regarding the number of covariates, the number of included studies, and the magnitude of effect modifiers. More specifically, we explored the following configurations:

- type of outcome: binary (B) and continuous (C).
- number of available covariates: 10, 15, of which 5, 8 were nuisance parameters, respectively.
- number of included studies: 5, 10.
- number of effect modifiers: 0 or 1 for the 10‐covariate scenarios and 2 or 3 for 15‐covariate scenarios.
- small vs large effect modification: details are shown in Section 2 of Appendix.
- heterogeneity of treatment effects (*τ*): 0.2 or 0.5.

These configurations amounted to 56 scenarios. In addition to the above, we explored 16 special scenarios under different assumptions.

- Four scenarios (Scenarios C29, C30, B29, B30) where all 10 available covariates were effect modifiers. Scenarios C29 and C30 had small effect modification. Scenarios B29 and B30 large effect modification.
- Four scenarios (Scenarios C31, C32, B31, B32) with nonnormal assumptions for the random effects. For Scenarios C31 and B31, random effects were drawn from Uniform(−0.4,0.4). For scenarios C32 and B32, random effects were drawn from Uniform(−1.0,1.0).
- Four scenarios (Scenarios C33, C34, B33, B34) where the variability of sample sizes in the studies was larger. We sampled the number of patients from Uniform(50,500).
- Four scenarios (Scenarios C35, C36, B35, B36) where there were 30 covariates, but only one effect modifier.

Section 2 of the Appendix summarizes all scenarios explored. For each scenario, we generated 1000 independent datasets. For each generated dataset, we used eight different models to perform the analysis. The first two models served as a reference. First, we fit a GLMM including only the covariates used to generate the simulated data (“GLMM‐oracle”). Second, we fit a GLMM including all covariates (“GLMM‐full”). We then used the variable selection or shrinkage models described in Section 2: STEP, LASSO, ridge, adaptive LASSO, Bayesian LASSO, and SSVS. In all models, we used common effects for effect modifiers. This means that although GLMM‐oracle contained the correct covariates, it was not identical to the data‐generating model. Table 1 summarizes all models we used in our simulation.

**TABLE 1:** Overview of the models used in the simulation study, including assumptions for the random effects structure

Model	Description	Variable selection	Shrinkage	Average treatment effect (*δ*)	Average main effects of covariates (*β*)	Average effect modification of covariates (*γ*)
GLMM‐full	All treatment‐covariate interactions are included in the model.	✘	✘	Random effect	Common effects	Common effects
GLMM‐oracle	The model only includes the main effects and treatment‐covariate interactions that were used to generate the simulated data in each scenario.	✘	✘	Random effect	Common effects	Common effects
STEP	Stepwise regression following bidirectional variable selection.	✔	✘	Common effect	Common effects	Common effects
LASSO	Frequentist model employing shrinkage on the covariates using an *L*_1_ penalty. Shrinkage is used only in treatment‐covariate interactions.	✔	✔	Common effect	Common effects	Common effects
ridge	Frequentist model employing shrinkage on the covariates using an *L*_2_ penalty. Shrinkage is used only in treatment‐covariate interactions.	✘	✔	Common effect	Common effects	Common effects
Adaptive LASSO	An extension of LASSO using adaptive weights.	✔	✔	Common effect	Common effects	Common effects
Bayesian LASSO	The Bayesian version of LASSO. Shrinkage is used only in treatment‐covariate interactions.	✘	✔	Random effect	Common effects	Common effects
SSVS	A Bayesian model that utilizes a mixture of priors to shrink only the treatment‐covariate interactions.	✘	✔	Random effect	Common effects	Common effects

*Note:* Note that in the data generating mechanisms for all scenarios, random effects were assumed across studies for coefficients ***β***, ***γ***, *δ* of Equation (1) and (2).

### 3.3 Measures of model performance

The usual goal of an IPD meta‐analysis is to estimate average treatment effects, to identify effect modifiers, and to estimate their impact on the outcome of interest. As a measure of performance, we assessed the deviation between the parameter estimates and their true values, using the mean squared error (MSE).

To assess the effect estimation of average treatment effects, we report the MSE for the treatment effect for zero values of all covariates, denoted *δ* in the data generating mechanism. To assess estimation of interactions, we calculated the MSE for the average interaction parameter *γ*_*m*_. We report the MSE for *γ*_*m*_ averaged across covariates that were actually effect modifiers (“true” effect modifiers), and we also report the MSE averaged across covariates that were not effect modifiers (“false” effect modifiers).

Because the ultimate aim is to estimate treatment effects at the patient level, we further report the MSE of the patient‐specific treatment effect, averaged over all simulated patients. More concretely, for each analysis model and for each simulated patient *i* we calculate the difference between the true treatment effect and the estimated treatment effect, that is, the difference between $\left(\hat{\gamma }\;x_{i}^{\mathrm{em}}+\hat{\delta }\right)$, where $x_{i}^{\mathrm{em}}$ are the effect modifiers that the model identified, and $\left(\gamma x_{i}^{\mathrm{EM}}+\delta \right)$, where $x_{i}^{\mathrm{EM}}$ are the true effect modifiers. For binary outcomes, MSEs are on the log odds ratio scale.

In addition, we report the SE of the average treatment effect and the average SE of the coefficients of the effect modifiers (continuous and binary averaged separately), for all methods except LASSO, ridge, and adaptive LASSO. As we discussed in Section 2.4, for LASSO there is no closed form solution for the SE. Although in ridge regression a closed form solution does exist for continuous outcomes, our model only penalizes ***γ*** and not ***α***_***j***_, *δ*, or ***β***. Standard ridge regression packages in R such as ridge ^33^ do not allow such choices, and standard errors cannot be obtained. A bootstrap method could be used to this end for LASSO, ridge, and adaptive LASSO. Bootstrapping each of the 1000 datasets that we generated per scenario would be computationally infeasible. Thus, we did not report standard errors for these three models.

Note that we did not compare models according to whether they correctly identified effect modifiers, for two reasons. First, ridge regression and the two Bayesian models do not perform variable selection in the strict sense of the term. Second, we deemed that the main aim of an IPD meta‐analysis is to obtain a precise estimate of patient‐specific treatment effects; we think that this was adequately captured by the measures of model performance described above.

### 3.4 Fitting details

All analyses were done in R.^34^ Before fitting the models we standardized both binary and continuous variables, that is, we transformed each one to a variable with mean of 0 and SD of 1. The standardization was necessary to ensure that penalization was equally applied to all regressors.^35^ For fitting GLMM‐oracle and GLMM‐full, we used the lme4 package.^36^ For STEP, we used a bidirectional selection model via the step function in the stats R package.^34^ We fitted LASSO, ridge, and adaptive LASSO using glmnet ^12^ and the *λ* value was chosen through a 10‐fold cross‐validation, using deviance as an error measure, where we compared 61 preselected *λ* values ranging from 0.001 to 1000. For adaptive LASSO, we used ${\hat{w}}_{m}=1/{\hat{\gamma }}_{m}$ where ${\hat{\gamma }}_{m}$ were obtained from the ridge regression.

We fitted Bayesian LASSO and SSVS using JAGS software through R. We used the rjags ^37^ and R2jags ^38^ packages which provide wrapper functions to implement the models in JAGS. To speed up the simulation process, we used the dclone package^39^ which allows parallel computing. For Bayesian LASSO, a vague prior was placed on *λ* in Equation (4). The prior we used was *Γ*(scale = 2, rate = 0.1); this has a mean of 20 and a variance of 200. We tested the sensitivity of results to the selection of prior distribution on *λ*, and found that the results did not materially change. For SSVS, we used the variant proposed by Meuwissen and Goddard,^32^ where *η*^2^ was assumed random (ie, *η* ∼ Uniform(0, 5)) and *g* was set to 100.

For all Bayesian methods, we ran two chains of 2000 iterations each, with 200 burn‐in iterations. We ensured that the number of iterations was sufficient to achieve convergence for all scenarios by performing preliminary checks using the Gelman‐Rubin diagnostics.^40^ For both models, we used a vague prior distribution *Γ*(scale = 0.001, rate = 0.001) for the precision of continuous outcomes 1/*σ*^2^; for the average treatment effect *δ*, average main effect *β*_*m*_, and study‐specific intercepts *α*_*j*_ we used a Normal(0, *σ*^2^ = 1000) noninformative prior.

The analysis of each scenario took from 5 hours up to 6 days to run on a laptop computer, depending on the complexity of the scenario. All codes used are available at https://github.com/MikeJSeo/phd/tree/master/shrinkage/simulation. In addition, we provide example code on how to fit all models in Section 4 of the Appendix, using mock datasets.

### 3.5 Simulation results

**FIGURE 1:**
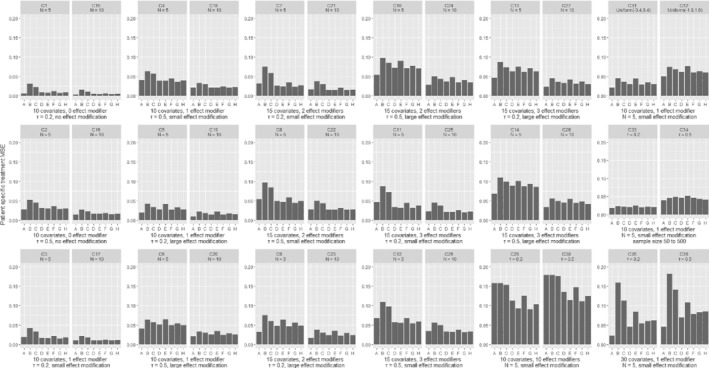
Results from simulations of a continuous outcome, comparing different methods in terms of the mean squared error for the patient‐specific treatment effect. Scenarios C1‐C28 are shown in pairs, differing only in the number of studies. Within each pair, the two scenarios explore the same type of outcome, have the same number of covariates and effect modifiers, equal heterogeneity and magnitude of effect modification. Scenarios C29‐C36 are shown in pairs, differing only in the assumed heterogeneity (*τ*). Scenarios are described in detail in Section 2 of the Appendix. A, GLMM‐oracle; B, GLMM‐full; C, STEP; D, LASSO; E, ridge; F, BAYES‐LASSO; G, SSVS

**FIGURE 2:**
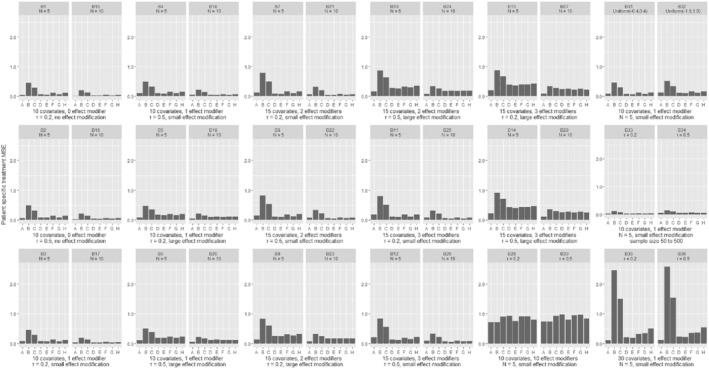
Results from simulations of a binary outcome, comparing different methods in terms of the mean squared error for the patient‐specific treatment effect. Scenarios B1‐B28 are shown in pairs, differing only in the number of studies. Within each pair, the two scenarios explore the same type of outcome, have the same number of covariates and effect modifiers, equal heterogeneity and magnitude of effect modification. Scenarios B29‐B36 are shown in pairs, differing only in the assumed heterogeneity (*τ*). Scenarios are described in detail in Section 2 of the Appendix. A, GLMM‐oracle; B, GLMM‐full; C, STEP; D, LASSO; E, ridge; F, BAYES‐LASSO; G, SSVS

**FIGURE 3:**
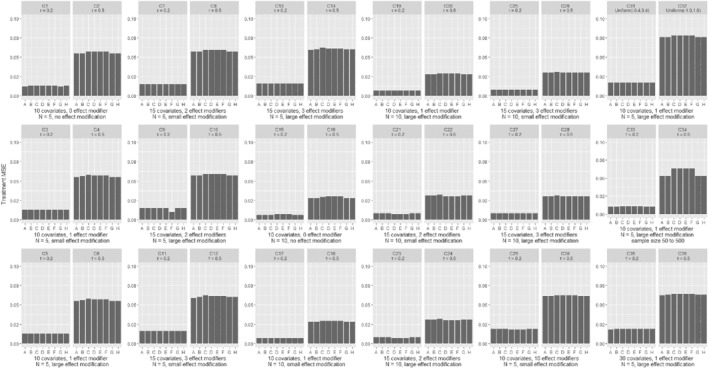
Results from simulations of a continuous outcome, comparing different methods in terms of the average treatment effect mean square error. Scenarios C1‐C28 are shown in pairs, differing only in the number of studies. Within each pair, the two scenarios explore the same type of outcome, have the same number of covariates and effect modifiers, equal heterogeneity and magnitude of effect modification. Scenarios C29‐C36 are shown in pairs, differing only in the assumed heterogeneity (*τ*). Scenarios are described in detail in Section 2 of the Appendix. A, GLMM‐oracle; B, GLMM‐full; C, STEP; D, LASSO; E, ridge; F, Adaptive LASSO; G, Bayesian LASSO; H, SSVS

**FIGURE 4:**
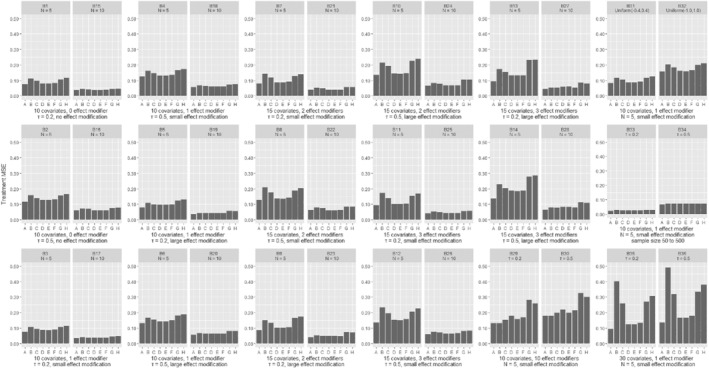
Results from simulations of a binary outcome, comparing different methods in terms of the average treatment effect mean square error. Scenarios (B1‐B28) are shown in pairs, differing only in the number of studies. Within each pair, the two scenarios explore the same type of outcome, have the same number of covariates and effect modifiers, equal heterogeneity and magnitude of effect modification. Scenarios B29‐B36 are shown in pairs, differing only in the assumed heterogeneity (*τ*). Scenarios are described in detail in Section 2 of the Appendix. A, GLMM‐oracle; B, GLMM‐full; C, STEP; D, LASSO; E, ridge; F, Adaptive LASSO; G, Bayesian LASSO; H, SSVS

In Figures 1 and 2, we present the comparison of the various models in terms of the estimation of patient‐specific treatment effects for continuous and binary outcomes; we deemed this to be the most important measure of performance in our assessment, and we discuss it more in detail in the following paragraphs. In Figures 3 and 4, we also present the MSE for estimation of the average treatment effect (ie, treatment effect at mean values of the covariates). We provide more detailed results from all simulation scenarios in Section 3 of the Appendix.

As shown in Figures 1 and 2, increasing the number of studies (while keeping all other parameters constant) led to better performance of all models. However, the number of studies did not affect the relative performance between the models. Likewise, increasing the heterogeneity of the average treatment effect (*τ*) generally led to worse performance, but did not affect the comparison between the models. As expected, GLMM‐oracle performed best in most scenarios. Besides GLMM‐oracle, we found that all shrinkage methods, that is, LASSO, ridge, adaptive LASSO, Bayesian LASSO, and SSVS performed better than models that do not use shrinkage, that is, GLMM‐full and STEP. In some scenarios (eg, C1, C7, B2, B11) the differences were quite pronounced. Furthermore, similar trend seems to hold for the special scenarios (ie, C31‐C36, B31‐B36). Especially in the scenario with large number of covariates with very few effect modifiers (ie, C35, C36), the benefit of shrinkage method is much more pronounced. For the readers' convenience, in Section 3 of the Appendix, Figures 1 and 2, we show again the patient‐specific treatment effect MSE, with scenarios grouped by heterogeneity of the treatment effect instead of the number of studies.

For both continuous and binary outcomes, we found that in most scenarios the differences between shrinkage methods were small. For binary outcomes the magnitude of MSEs was much larger. Rather surprisingly, there were some scenarios where GLMM‐oracle did not outperform other methods.

Figures 3 and 4 show the MSE for the estimation of average treatment effect across different models. For continuous outcomes, there were almost no differences across the competing models. The frequentist methods with shrinkage performed slightly better for binary outcomes. Section 3 of the Appendix shows the results in terms of true effect modifier MSE. When the effect modification was small, the true effect modifier MSE was lower for shrinkage methods. When the effect modification was large, shrinkage methods had higher MSE. In Section 3 of the Appendix, we also show the false effect modifier MSE, which was always lower for the shrinkage models.

Finally, the SEs in the estimation of coefficients of effect modifiers were similar, but SEs for treatment effects varied across models. Overall, Bayesian models had the highest SEs and STEP had the lowest. In Bayesian models, the vague priors assumed for *τ* often overestimated the heterogeneity. Using a more informative prior would have lowered the SE. Furthermore, although the STEP model had the lowest standard error, this is mainly because model uncertainty (ie, regarding which interactions to include) was not considered in calculating the standard errors. In other words, estimation of standard errors for STEP was performed as if the reduced model was prespecified.^41^ Moreover, as discussed in the previous section, there was no easy way of calculating standard errors for LASSO, ridge, and adaptive LASSO. Thus, standard errors of the estimated quantities were not ideal for comparing the performance of the different methods.

## 4 REAL DATASETS

In this section, we describe two real datasets that we used to illustrate the methods we described in the previous paragraphs.

### 4.1 Newer generation drug‐eluting or bare‐metal stents for percutaneous coronary intervention

The dataset comprises IPD from eight RCTs in 11 133 patients who underwent percutaneous coronary intervention for a coronary artery disease. The RCTs compared new generation drug‐eluting with bare metal stents. The outcome in this analysis is a composite binary outcome, that is, cardiac death or myocardial infarction at 1‐year after randomization. The dataset contains several patient‐level covariates, including one continuous covariate (age), one count covariate (number of implanted stents), and seven binary covariates (gender, diabetes, clinical presentation at the time of percutaneous coronary intervention, multivessel disease, target vessel left anterior descending artery, overlapping stents, and mean stent diameter ≥ 3 mm).^42^ Section 1 of the Appendix describes outcome, treatment, and covariates for complete case participants and participants with at least one missing covariate.

### 4.2 Antidepressant treatment of major depression

The dataset comprises IPD from four placebo‐controlled RCTs on 1261 patients. The trials examined the effects of antidepressant treatments in acute major depression. The outcome of interest is depression severity on a continuous scale at week 6 or 8. Patient‐level covariates include two binary covariates (sex and episode frequency dichotomized at greater than or equal to three episodes) and nine continuous covariates (baseline severity, age, age at onset, episode duration, and the 17‐item Hamilton rating scale for depression consisting of five subscales covering anhedonia, guilt, bodily symptoms, appetite, and insomnia).^43^ Section 1 of the Appendix includes descriptions for this dataset.

## 5 APPLICATION IN REAL DATASETS

### 5.1 Newer generation drug‐eluting vs bare‐metal stents

Table 2 shows the results from all models fitted to the data described in Section 4.1. Age, diabetes, clinical presentation at the time of percutaneous coronary intervention, multivessel disease, and overlapping stents were the most important prognostic factors. Overall, there was little evidence of effect modification for most covariates. The covariate with the strongest effect modification was target vessel left anterior descending artery vs other vessel (“ladtreated” in Table 2). This covariate was selected in STEP, LASSO, and adaptive LASSO, was included in 70% of the SSVS iterations, and had a relatively large coefficient in all models.

**TABLE 2:** Results from fitting various models in the stents dataset

Parameter		GLMM‐full (SE)	STEP (SE)	LASSO (SE*)	ridge (SE*)	Adaptive LASSO (SE*)	Bayesian LASSO (SE)	SSVS (SE, % selected)
Average treatment effect (log‐odds ratio at mean values of the covariates)		0.04 (0.12)	−0.05 (0.09)	−0.07 (0.11)	−0.05 (0.11)	−0.05 (0.11)	−0.04 (0.14)	−0.03 (0.15)
Heterogeneity (*τ*)		0.00	–	–	–	–	0.20 (0.16)	0.21 (0.18)
Main effects	age	0.70 (0.09)	0.65 (0.06)	0.65 (0.08)	0.66 (0.08)	0.65 (0.08)	0.67 (0.07)	0.67 (0.07)
	gender	−0.01 (0.06)	0.00 (0.04	0.00 (0.04)	−0.00 (0.04)	0.00 (0.04)	−0.01 (0.05)	−0.00 (0.05)
	diabetes	0.20 (0.06)	0.18 (0.04)	0.18 (0.04)	0.19 (0.05)	0.18 (0.04)	0.19 (0.05)	0.19 (0.05)
	stable_cad	−0.26 (0.07)	−0.24 (0.05)	−0.24 (0.05)	−0.24 (0.05)	−0.24 (0.06)	−0.25 (0.06)	−0.24 (0.06)
	multivessel	0.15 (0.07)	0.10 (0.05)	0.11 (0.07)	0.12 (0.06)	0.13 (0.07)	0.13 (0.06)	0.13 (0.06)
	ladtreated	0.10 (0.07)	0.10 (0.06)	0.05 (0.06)	0.03 (0.05)	0.09 (0.06)	0.04 (0.06)	0.05 (0.07)
	overlap	0.15 (0.06)	0.14 (0.05)	0.14 (0.06)	0.14 (0.06)	0.14 (0.06)	0.15 (0.05)	0.15 (0.05)
	m_dia_above_3	−0.05 (0.05)	−0.01 (0.04)	−0.02 (0.05)	−0.02 (0.05)	−0.04 (0.05)	−0.02 (0.04)	−0.02 (0.04)
	num_stents	0.02 (0.07)	0.04 (0.06)	0.00 (0.06)	−0.00 (0.05)	0.02 (0.06)	−0.00 (0.06)	−0.00 (0.06)
Effect modifiers (treatment‐covariate interactions)	age: treatment	−0.08 (0.11)	0	0	−0.02 (0.05)	0	−0.03 (0.06)	−0.04 (0.07, 48%)
	gender: treatment	0.02 (0.08)	0	0	0.01 (0.04)	0	0.01 (0.05)	0.01 (0.04, 37%)
	diabetes: treatment	−0.03 (0.08)	0	0	−0.01 (0.04)	0	−0.01 (0.05)	−0.01 (0.04, 38%)
	stable_cad: treatment	0.05 (0.10)	0	0	0.02 (0.04)	0	0.02 (0.05)	0.01 (0.05, 40%)
	multivessel: treatment	−0.09 (0.09)	0	−0.00 (0.06)	−0.03 (0.05)	−0.05 (0.07)	−0.04 (0.06)	−0.04 (0.07, 50%)
	ladtreated: treatment	−0.18 (0.09)	−0.20 (0.09)	−0.10 (0.09)	−0.06 (0.07)	−0.16 (0.10)	−0.08 (0.07)	−0.09 (0.09, 70%)
	overlap: treatment	−0.02 (0.09)	0	0	−0.02 (0.04)	0	−0.02 (0.05)	−0.02 (0.05, 39%)
	m_dia_above_3: treatment	0.09 (0.07)	0	0.03 (0.07)	0.04 (0.05)	0.07 (0.07)	0.04 (0.05)	0.04 (0.05, 47%)
	num_stents: treatment	−0.08 (0.10)	−0.12 (0.08)	−0.04 (0.06)	−0.04 (0.04)	−0.08 (0.07)	−0.04 (0.06)	−0.04 (0.06, 49%)

*Note:* “age: treatment” denotes the interaction term between age and treatment; likewise for other covariates. For SSVS, the “% selected” shows how frequently a given interaction was selected in the MCMC analysis. stable_cad: stable coronary artery disease (clinical presentation at the time of percutaneous coronary intervention); ladtreated: target vessel left anterior descending artery; m_dia_above_3: mean diameter ≥ 3 mm; num_stents: number of implanted stents. SE, standard error. SE denoted with a star (*) were estimated using bootstrap.

Our readers should note that the results presented in Table 2 may not be very informative for clinical practice. For clinicians, the estimated relative treatment effects for clinically relevant patient subgroups would be more valuable. For instance, using the Bayesian LASSO model, for the subgroup of patients aged 80 years, female, with diabetes, unstable coronary artery disease, multivessel disease, target vessel left anterior descending artery, overlapping stents, mean stent diameter < 3 mm, and 5 stents, we estimated an odds ratio of 0.54 (95% CrI 0.24, 1.18). This means that patients in this subgroup were estimated to have 46% lower odds of cardiac death or myocardial infarction when treated with newer generation drug‐eluting stents. On the other hand, for patients aged 50 years old, male, with no diabetes, stable coronary artery disease, no multivessel disease, no target vessel left anterior descending artery, no overlapping stents, mean stent diameter ≥ 3, and 1 stent, the estimated odds ratio was 1.22 (95% CrI 0.75, 2.00). Thus, the model estimated a 22% increase in the odds of dying from cardiac death or having a myocardial infarction. Although both credible intervals are quite wide, we conclude that (all other things being equal) the treatment should be assigned to the first group rather than to the second. Of note, newer generation drug‐eluting stents are much more expensive than bare‐metal stents, and giving priority to the first group may be cost‐effective. The results for these two patient subgroups from all models are shown in Table 3. It is interesting to note that GLMM‐full gave impressively large estimates of relative effects: odds ratio 0.28 (95% CrI 0.11, 0.73) for the first, and 1.84 (0.98, 3.45) for the second subgroup. Based on the findings of our simulations, we conclude that they are probably an overestimation of the true treatment effects.

**TABLE 3:** Estimated treatment effect (odds ratio and 95% CI) for different subgroup population in Stent dataset

Subgroups	GLMM‐full (95% CI)	STEP (95% CI)	LASSO (95% CI*)	ridge (95% CI*)	Adaptive LASSO (95% CI*)	Bayesian LASSO (95% CrI)	SSVS (95% CrI)
80 years old, female, diabetes, unstable CAD, multivessel disease, ladtreated, overlapping stents, mean stent diameter < 3 mm, and 5 stents	0.28 (0.11, 0.73)	0.52 (0.32, 0.86)	0.63 (0.17, 0.98)	0.43 (0.18, 0.94)	0.42 (0.15, 0.97)	0.54 (0.24, 1.18)	0.55 (0.24, 1.27)
50 years old, male, no diabetes, stable CAD, no multivessel disease, no ladtreated, no overlapping stents, mean stent diameter ≥ 3 mm, and 1 stent	1.84 (0.98, 3.45)	1.24 (0.94, 1.63)	1.05 (0.80, 2.61)	1.32 (0.84, 2.27)	1.26 (0.84, 2.52)	1.22 (0.75, 2.00)	1.24 (0.73, 2.11)

*Note:* All other abbreviations as per Table 2. CI, confidence interval; CrI, credible interval. CI denoted with a star (*) were estimated using bootstrap. An odds ratio smaller than 1 favors the drug eluding stents.

Finally, from a practical, clinical point of view, we note that it would be useful to provide clinicians with a tool (eg, an online calculator or application) that will allow them to enter patient characteristics and obtain an estimate of relative effectiveness of the intervention. This however falls beyond the aims of this article.

### 5.2 Antidepressants

Table 4 shows the results from all models fitted to the data described in Section 4.2. Important prognostic factors included baseline severity, age, age at onset, episode duration, bodily symptoms (Hamilton score), and anhedonia (Hamilton score). There was no evidence of effect modification for most covariates. The covariate with the strongest interaction with treatment was age at onset. This covariate was selected in STEP, LASSO, and adaptive LASSO, was included in 81% of the SSVS iterations, and had a relatively large coefficient in all models.

**TABLE 4:** Results from fitting various models in the antidepressant dataset

Parameter		GLMM‐full (SE)	STEP (SE)	LASSO (SE*)	ridge (SE*)	Adaptive LASSO (SE*)	Bayesian LASSO (SE*)	SSVS (SE, % selected)
Average treatment effect (log‐odds ratio)		−1.37 (0.35)	−1.37 (0.35)	−1.36 (0.30)	−1.27 (0.31)	−1.37 (0.89)	−1.38 (0.51)	−1.34 (0.51)
Heterogeneity (*τ*)		0.00	–	–	–	–	0.57 (0.44)	0.55 (0.44)
Main effects	baseline severity	1.65 (1.23)	1.94 (0.67)	1.95 (0.82)	1.94 (0.80)	1.95 (0.77)	1.90 (0.73)	1.99 (0.69)
	age	1.08 (0.74)	0.55 (0.44)	0.56 (0.62)	0.49 (0.67)	0.55 (0.67)	0.55 (0.54)	0.56 (0.49)
	female	0.10 (0.30)	−0.22 (0.16)	−0.19 (0.27)	−0.14 (0.26)	−0.45(0.52)	−0.00 (0.27)	−0.12 (0.24)
	age at onset	−1.97 (0.74)	−1.50 (0.48)	−1.25 (0.64)	−1.03 (0.71)	−1.37 (0.80)	−1.39 (0.56)	−1.26 (0.53)
	episode_frequency_over_3	0.03 (0.37)	−0.02 (0.19)	−0.03 (0.31)	0.03 (0.30)	−0.02 (0.34)	0.08 (0.30)	0.02 (0.25)
	episode duration week	0.46 (0.32)	0.50 (0.17)	0.49 (0.24)	0.49 (0.23)	0.49 (0.25)	0.47 (0.27)	0.49 (0.21)
	guilty agitation HRSD	−0.07 (0.63)	−0.02 (0.35)	−0.01 (0.43)	−0.04 (0.41)	−0.01 (0.41)	−0.10 (0.41)	−0.08 (0.38)
	bodily symptoms HRSD	0.64 (0.52)	0.29 (0.28)	0.28 (0.37)	0.33 (0.34)	0.28 (0.32)	0.45 (0.38)	0.32 (0.33)
	sleep problems HRSD	0.22 (0.59)	0.12 (0.32)	0.12 (0.33)	0.11 (0.34)	0.12 (0.41)	0.12 (0.39)	0.10 (0.34)
	anhedonia retardation HRSD	0.32 (0.60)	0.34 (0.32)	0.33 (0.43)	0.29 (0.42)	0.33 (0.45)	0.27 (0.39)	0.27 (0.36)
Effect modifiers (treatment‐covariate interactions)	baseline severity: treatment	0.52 (1.47)	0	0	0.03 (0.37)	0	0.09 (0.44)	0.02 (0.24, 40%)
	age: treatment	−0.78 (0.92)	0	0	0.15 (0.67)	0	0.06 (0.46)	0.07 (0.32, 46%)
	female: treatment	−0.45 (0.35)	0	−0.05 (0.35)	−0.13 (0.32)	0	−0.31 (0.31)	−0.15 (0.25, 47%)
	age at onset: treatment	1.69 (0.92)	0.99 (0.33)	0.60 (0.65)	0.23 (0.74)	0.81 (0.99)	0.76 (0.52)	0.56 (0.48, 81%)
	episode frequency over 3: treatment	−0.04 (0.43)	0	0	−0.09 (0.26)	0	−0.15 (0.31)	−0.07 (0.21, 37%)
	episode duration week: treatment	0.06 (0.37)	0	0	−0.01 (0.20)	0	0.02 (0.29)	0.00 (0.18, 35%)
	guilty agitation HRSD: treatment	0.01 (0.76)	0	0	0.04 (0.26)	0	0.10 (0.33)	0.06 (0.21, 39%)
	bodily symptoms HRSD: treatment	−0.52 (0.62)	0	0	−0.07 (0.31)	0	−0.24 (0.33)	−0.09 (0.23, 41%)
	sleep problems HRSD: treatment	−0.20 (0.70)	0	0	−0.00 (0.20)	0	−0.02 (0.31)	−0.01 (0.18, 35%)
	anhedonia retardation HRSD: treatment	−0.01 (0.71)	0	0	0.06 (0.26)	0	0.10 (0.32)	0.06 (0.20, 38%)

*Note:* “age: treat” denotes the interaction term between age and treatment; likewise for all other interaction terms. For SSVS, the “% selected” shows how frequently a given interaction was selected in the MCMC analysis. HRSD, 17‐item Hamilton rating scale for depression; SE, standard error. SE denoted with a star (*) were estimated using bootstrap.

In Table 5, we report the estimated relative treatment effects for two different patient subgroups. Using Bayesian LASSO for the subgroup of patients with baseline severity of 15, aged 25 years, female sex, onset at age 20 years, episode frequency ≥ 3, episode duration of 42 weeks, Hamilton score for guilt 5, for bodily symptoms 5, sleeping problems 5, and anhedonia retardation 7, we estimated a relative effect of −2.9 (95% CrI of −5.6 to −0.3) favoring antidepressants, a clinically meaningful result. On the other hand, for the subgroup of patients with baseline severity of 25, aged 45 years, male, onset at age 45 years, episode frequency < 3, episode duration of 42 weeks, Hamilton score for guilt 5, for bodily symptoms 2, sleep problems 1, and anhedonia retardation 7, the estimated relative treatment was 0.8 with 95% CrI of −1.9 to 3.5.

**TABLE 5:** Estimated treatment effect (difference in Hamilton rating scale for depression, HRSD) for different subgroup population in the antidepressant dataset

Subgroups	GLMM‐full (95% CI)	STEP (95% CI)	LASSO (95% CI*)	ridge (95% CI*)	Adaptive LASSO (95% CrI)	Bayesian LASSO (95% CrI)	SSVS (95% CrI)
Baseline severity of 15, 25 years old, female, onset at age 20, episode frequency ≥ 3, episode duration of 42 weeks, guilty agitation HRSD = 5, bodily symptoms HRSD = 5, sleep problems HRSD = 5, anhedonia retardation HRSD = 7	−3.7 (−10.2, 2.9)	−2.7 (−3.8, −1.6)	−2.1 (−5.0, −0.7)	−2.0 (−4.6, −0.8)	−2.6 (−5.1, −0.8)	−2.9 (−5.6, −0.3)	−2.2 (−4.2, −0.2)
Baseline severity of 25, 45 years old, male, onset at age 45, episode frequency < 3, episode duration of 42 weeks, guilty agitation HRSD = 5, bodily symptoms HRSD = 2, sleep problems HRSD = 1, anhedonia retardation HRSD = 7	2.5 (−3.0, 8.0)	−0.3 (−1.3, 0.7)	−0.6 (−1.7, 3.6)	−0.5 (−1.5, 4.4)	−0.1 (−1.6, 5.3)	0.8 (−1.9, 3.5)	−0.2 (−2.4, 2.0)

*Note:* CI, confidence interval; CrI, credible interval. CI denoted with a star (*) were estimated using bootstrap. A treatment effect less than zero favors antidepressants over placebo.

### 5.3 Fitting details

For both datasets (stents, antidepressants) fitting details were the same as described in the simulations section. The only difference was that for the Bayesian methods, we used three chains of 10 000 iterations with 1000 burn‐in. The R code we used for fitting all models are available at https://github.com/MikeJSeo/phd/tree/master/shrinkage/. Furthermore, the R package bipd available at https://github.com/MikeJSeo/bipd implements the two Bayesian models (ie, Bayesian LASSO and SSVS) discussed in our article. The package contains prebuilt functions to produce JAGS code, run JAGS code with or without parallelization, and calculate measures such as the patient‐specific treatment effect mentioned in this article. The code in the appendix demonstrates how to use this package in practice.

## 6 DISCUSSION

We explored different methods for estimating patient‐specific treatment effects in IPD meta‐analysis. In detailed simulations, we compared the performance of stepwise regression and five shrinkage methods (frequentist LASSO, ridge, and adaptive LASSO, Bayesian LASSO, and SSVS) with the standard approach of including all available variables in a generalized linear mixed model. In our simulations, we investigated various scenarios, for continuous and binary outcomes, for different numbers of covariates, included studies, effect modifiers, amount of heterogeneity of treatment effects, and the extent of effect modification. We also applied all models to two clinical examples, to illustrate their use in practice.

We found that, overall, methods with shrinkage on effect modifiers (ie, LASSO, ridge, adaptive LASSO, Bayesian LASSO, and SSVS) performed better (ie, gave better estimates of patient‐specific treatment effects) than the standard approach of including all covariates in the model (GLMM‐full) and stepwise regression. The added benefit of shrinkage methods was large in some scenarios, for both continuous and binary outcomes. Furthermore, we found that the four shrinkage methods performed comparably well in most cases. Using two clinical examples we illustrated that shrinkage methods can give substantially different estimates as compared with nonshrinkage models, for specific patient subgroups.

Several limitations are worth mentioning. First, we could have improved the performance of STEP by using postestimation shrinkage after fitting, that is, by multiplying the regression coefficients with a global shrinkage factor, as done in the package shrink, by Dunkler et al.^44^ One disadvantage of using this approach is that this shrinks the coefficients globally, while in our applications, we only shrink the effect modifiers. Thus, we did not explore such extensions of STEP in this article. Moreover, we have not considered the best subset regression method. This method tests all possible combinations of the predictor variables, and selects the best model according to some measure, for example, adjusted R‐squared or Mallow's *C*_*p*_.^45^ To the best of our knowledge, the currently available packages that implement this algorithm only allow continuous outcomes, and we did not pursue this further.^46^ Another model we did not use is the generalized linear mixed effects model using *L*_1_‐penality (GLMM‐LASSO)^47^; that is, we only employed the common effects LASSO. Exploratory analyses showed that the implementation of GLMM‐LASSO using the existing package in R is problematic, as estimates were heavily influenced by the choice of starting values. Thus, we decided to exclude this model from our comparison.

As mentioned, when using shrinkage models we only penalized the coefficients for effect modifiers, and left main effects without penalization. Otherwise, models such as LASSO or STEP could have dropped the main effects of a variable while keeping the interaction term. We deemed that this would lead to unrealistic models, and we decided not to explore this further. Furthermore, when finding the best fitting *λ* for LASSO or RIDGE, we considered 10‐fold cross‐validation that randomly selected patients without accounting for clustering in studies. Although improbable, this might lead to computational errors if all patients of a study were selected for the same fold (because *a*_*j*_ would not be estimable from the rest). A possible solution to this would be to select a fixed proportion of patients from each study in each fold.

In addition, we only used common effects for prognostic factors and effect modifiers when fitting all models (although in simulations the data generating mechanisms assumed heterogeneity in both). This was done because (a) estimating many random effects is difficult, especially for small number of studies, (b) some of the frequentist methods with random *γ*_*k*_ are not available for easy application in R, and (c) initial analyses showed that for the Bayesian models it is often difficult to estimate the heterogeneity of an effect modifier ($\tau_{\gamma ,m}^{2}$) while also shrinking the coefficient of the corresponding covariate. In such situations, $\tau_{\gamma ,m}^{2}$ may acquire large posterior values and the estimates of the coefficients are unduly shrank to zero. This problem could be resolved by using informative priors (narrowly distributed near zero) for all $\tau_{\gamma ,m}^{2}$. Alternatively, we could use a more complicated approach, such as allowing the random effects to drop out of the model with the use of mixture priors for $\tau_{\gamma ,m}^{2}$.^48^, ^49^ However, we did not investigate this in more detail. Further exploration of this issue might be an interesting future research topic.

We only used noninformative prior distributions for all parameters in our Bayesian models. For instance, for the SSVS, we assumed that each variable has 0.5 a priori chance of entering the model. Boulet et al,^16^ showed how clinical relevance weights can be incorporated for each variable. Incorporating such an informative prior might lead to an improved performance of SSVS. Similarly, we could improve the performance of Bayesian LASSO by using informative priors for some parameters, for example, heterogeneity of treatment effects.^50^, ^51^

We did not disentangle within‐ from across‐trial interactions in our models. Although we did not simulate study‐level interactions in our simulations, using this approach runs the risk of aggregation bias in clinical applications. To avoid this bias, it has been recommended that interaction effects should be estimated using within‐trials information alone.^52^, ^53^ In practice, meta‐analysts are advised to check whether the interaction estimates after amalgamating within‐ and across‐trial information are different in magnitude from the estimates of using within‐trial information alone. If the difference is large, the latter approach may not give an accurate picture of treatment effect modification. One caveat with using within‐trials information alone is that it is not obvious how to use such a model to make recommendations for new patients in real clinical practice, as it needs the “study‐specific” average of all covariates, as an input for a new patient. We did not explore this further in this article, but it might be an interesting area for future research.

Throughout this article, we assumed complete datasets for simplicity. However, analysis using missing data imputation is possible for all of the models we considered, including multiple imputation as the standard approach to addressing missing data.^54^, ^55^ For Bayesian methods, fully model‐based approaches can also be used, where missing values are imputed within each iteration of the MCMC. Future research could explore the performance of different variable selection or shrinkage methods depending on data availability and missingness mechanisms. Another area of possible future research is to examine how our findings generalize to multiple treatments, that is, in a network meta‐analysis.^56^

Another limitation of our simulation is that we assumed a similar case‐mix in the simulated studies. This is probably an oversimplification, that is, we expect in practice different studies to have differences in case‐mix. Moreover, the linear models that we used are generally crude simplifications of the true underlying mechanisms, where we expect to find nonlinear relationships, interactions between covariates, and so on. Future work might be focused on exploring the performance of all methods in such scenarios.

In this article, we used simulations to try to identify which of the competing methods works best under various data generating mechanisms. However, in real applications of IPD meta‐analysis the generating mechanisms are unknown, while it is not straightforward to measure model performance in order to decide between competing models. To be more precise, our models do not predict the outcome; thus, we cannot compare predictions with an observed value in order to assess the models' accuracy. Instead, our models estimate relative treatment effects, that is, the potential benefit of treatment A vs B. Since each patient only takes either A or B (in usual parallel RCT designs) the treatment benefit is unobservable.

Finally, often IPD meta‐analysis is conducted in a decentralized way, due to data privacy issues. This happens when data from different studies cannot be merged, and thus are analyzed separately and results are pooled on a second stage. All methods (with or without shrinkage) described in this article might in principle be used for a two‐stage analysis after some modifications; however, we did not explore this further. This might be interesting as a follow‐up project.

This is the first article to compare variable selection and shrinkage methods formally in the context of IPD meta‐analysis, and its findings should be valuable for informing the conduct of future IPD meta‐analyses. In practice, we recommend that meta‐analysts use shrinkage methods. The choice between the different shrinkage methods will be influenced by the demands on computational time (frequentist methods are much easier to fit) and by the expertise of the meta‐analysts in the various statistical packages (Bayesian methods require familiarity with specialized software such as JAGS). When possible, we recommend the use of Bayesian shrinkage models over the frequentist ones. The Bayesian models allow for greater flexibility, for example, in setting up random effect structures, and allow incorporating prior information regarding some of the model's parameters.

In summary, we found that methods for IPD meta‐analysis that perform shrinkage on treatment‐covariate interactions estimate patient‐specific treatment effects better than the standard approach of including all covariates in the model, and better than stepwise regression. Depending on the clinical settings, the added benefit of the shrinkage methods can be quite substantial.

## CONFLICT OF INTEREST

Toshi A. Furukawa reports personal fees from Mitsubishi‐Tanabe, MSD and Shionogi, and a grant from Mitsubishi‐Tanabe, outside the submitted work; Toshi A. Furukawa has a patent 2018‐177688 pending.

## Supporting information


**Appendix S1**: supporting information

## Data Availability

No new data were created in this study. The data we used for both clinical examples were provided by the original data owners, and are not publicly available.
