# Supplementary material for: Comparing methods for estimating patient‐specific treatment effects in individual patient data meta‐analysis
Source: Stat Med. 2020 Dec 27;40(6):1553–73. doi: 10.1002/sim.8859 (PMC7898845; doi:10.1002/sim.8859)
Supplement: Supplementary file 1 — Appendix S1: supporting information [file SIM-40-1553-s001.docx]

**Appendix for “Comparing methods for estimating patient-specific treatment effects in individual patient data meta-analysis”**

Michael Seo^1,2^, Ian R. White^3^, Toshi A. Furukawa^4^, Hissei Imai^4^, Marco Valgimigli^5^, Matthias Egger^1^, Marcel Zwahlen^1^, Orestis Efthimiou^1^

*^1^ Institute of Social and Preventive Medicine, University of Bern, Switzerland*

*^2^ Graduate School for Health Sciences, University of Bern, Switzerland*

*^3^ MRC Clinical Trials Unit, Institute of Clinical Trials and Methodology, University College London,
London, UK*

*^4^* *Departments of Health Promotion and Human Behavior and of Clinical Epidemiology, Kyoto University Graduate School of Medicine / School of Public Health, Kyoto, Japan*

*^5^* *Department of Cardiology, Bern University Hospital, University of Bern, Switzerland*

# Descriptive statistics of the real datasets used in the paper

Table 1: Stents datasets. Separate summary is reported below for complete cases patients and patients with at least one missing covariate. stable_cad: stable coronary artery disease (clinical presentation at the time of percutaneous coronary intervention); ladtreated: target vessel left anterior descending artery; m_dia_above_3: mean diameter ≥ 3 mm; num_stents: number of implanted stents.

| **Variable** | **Complete cases (n = 11106)** | **Patients with at least one missing covariate (n =327)** |
| --- | --- | --- |
|  | **Mean(SD) for continuous or % for binary** | **Mean(SD) for continuous or % for binary** |
| binary outcome: cardiac death or myocardial infarction at 1-year after randomization | 5% | 7% |
| treatment (drug eluding stents) | 56% | 54% |
| age | 68.60 (12.22) | 70.47 (12.09) |
| gender | 73% | 78% |
| diabetes | 23% | 28% |
| stable_cad | 34% | 35% |
| multivessel | 46% | 52% |
| ladtreated | 47% | 48% |
| overlap | 15% | 12% |
| m_dia_above_3 | 97% | 96% |
| num_stents | 1.66 (1.05) | 1.26 (0.77) |

Table 2: Antidepressant dataset. Separate summary is reported below for complete cases patients and patients with at least one missing covariate. HRSD: 17-item Hamilton Rating Scale for Depression

| **Variable** | **Complete cases (n =1261)** | **Patients with at least one missing covariate (n =232)** |
| --- | --- | --- |
|  | **Mean(SD) for continuous or % for binary** | **Mean(SD) for continuous or % for binary** |
| continuous outcome: depression severity at week 6 or 8 | 9.66 (6.19) | 6.50 (4.41) |
| treatment (antidepressants) | 71% | 69% |
| baseline severity | 20.92 (4.38) | 21.70 (4.29) |
| age | 36.72 (9.93) | 39.48 (12.05) |
| female | 50% | 50% |
| age at onset | 33.58 (10.40) | 35.50 (12.37) |
| episode frequency over 3 | 16% | 22% |
| episode duration week | 42.04 (55.73) | 53.53 (102.56) |
| guilty agitation HRSD | 5.48 (2.06) | 5.43 (2.12) |
| bodily symptoms HRSD | 3.68 (1.50) | 3.98 (1.59) |
| sleep problems HRSD | 3.10 (1.68) | 3.26 (1.65) |
| anhedonia retardation HRSD | 7.48 (1.71) | 7.65 (1.81) |

# Presentation of scenarios explored in simulations

In the following table, we present an overview of the scenarios we explored in our simulation study.

Table 3: Overview of the scenarios we explored in our simulations. In the column with the true values for effect modifier, we first present the main effect on the outcome and in the parenthesis the interaction with the treatment (i.e. effect modification). Scenario C1 models continuous outcome and scenario B1 models binary outcome.

| **Scenarios** | **# of studies** | **# of covariates** | **# of nuisance covariates** | **# of prognostic factors** | **# of effect modifiers** | **True values for effect modifiers: main effect**  **(effect modification)** | **Heterogeneity of treatment effect** |
| --- | --- | --- | --- | --- | --- | --- | --- |
| C1 | 5 | 10 | 3 continuous  2 binary | 3 continuous  2 binary | None | NA | $\tau$=0.2 |
| C2 |  |  |  |  |  |  | $\tau$=0.5 |
| C3 | 5 | 10 | 3 continuous  2 binary | 2 continuous  2 binary | 1 continuous | continuous: 0.2 (0.1) | $\tau$=0.2 |
| C4 |  |  |  |  |  |  | $\tau$=0.5 |
| C5 | 5 | 10 | 3 continuous  2 binary | 2 continuous  2 binary | 1 continuous | continuous: 0.2 (0.5) | $\tau$=0.2 |
| C6 |  |  |  |  |  |  | $\tau$=0.5 |
| C7 | 5 | 15 | 5 continuous  3 binary | 3 continuous  2 binary | 1 continuous  1 binary | continuous: 0.2 (0.1)  binary: 0.2 (0.1) | $\tau$=0.2 |
| C8 |  |  |  |  |  |  | $\tau$=0.5 |
| C9 | 5 | 15 | 5 continuous  3 binary | 3 continuous  2 binary | 1 continuous  1 binary | continuous: 0.2 (0.5)  binary: 0.2 (0.4) | $\tau$=0.2 |
| C10 |  |  |  |  |  |  | $\tau$=0.5 |
| C11 | 5 | 15 | 5 continuous  3 binary | 2 continuous  2 binary | 2 continuous  1 binary | continuous: 0.2 (0.1)  binary: 0.2 (0.1) | $\tau$=0.2 |
| C12 |  |  |  |  |  |  | $\tau$=0.5 |
| C13 | 5 | 15 | 5 continuous  3 binary | 2 continuous  2 binary | 2 continuous  1 binary | continuous: 0.2 (0.5)  binary: 0.2 (0.4) | $\tau$=0.2 |
| C14 |  |  |  |  |  |  | $\tau$=0.5 |
| C15 | 10 | 10 | 3 continuous  2 binary | 3 continuous  2 binary | None | NA | $\tau$=0.2 |
| C16 |  |  |  |  |  |  | $\tau$=0.5 |
| C17 | 10 | 10 | 3 continuous  2 binary | 2 continuous  2 binary | 1 continuous | continuous: 0.2 (0.1) | $\tau$=0.2 |
| C18 |  |  |  |  |  |  | $\tau$=0.5 |
| C19 | 10 | 10 | 3 continuous  2 binary | 2 continuous  2 binary | 1 continuous | continuous: 0.2 (0.5) | $\tau$=0.2 |
| C20 |  |  |  |  |  |  | $\tau$=0.5 |
| C21 | 10 | 15 | 5 continuous  3 binary | 3 continuous  2 binary | 1 continuous  1 binary | continuous: 0.2 (0.1)  binary: 0.2 (0.1) | $\tau$=0.2 |
| C22 |  |  |  |  |  |  | $\tau$=0.5 |
| C23 | 10 | 15 | 5 continuous  3 binary | 3 continuous  2 binary | 1 continuous  1 binary | continuous: 0.2 (0.5)  binary: 0.2 (0.4) | $\tau$=0.2 |
| C24 |  |  |  |  |  |  | $\tau$=0.5 |
| C25 | 10 | 15 | 5 continuous  3 binary | 2 continuous  2 binary | 2 continuous  1 binary | continuous: 0.2 (0.1)  binary: 0.2 (0.1) | $\tau$=0.2 |
| C26 |  |  |  |  |  |  | $\tau$=0.5 |
| C27 | 10 | 15 | 5 continuous  3 binary | 2 continuous  2 binary | 2 continuous  1 binary | continuous: 0.2 (0.5)  binary: 0.2 (0.4) | $\tau$=0.2 |
| C28 |  |  |  |  |  |  | $\tau$=0.5 |
| C29 | 5 | 10 | None | None | 5 continuous  5 binary | continuous: 0.2 (0.1)  binary: 0.2 (0.1) | $\tau$=0.2 |
| C30 |  |  |  |  |  |  | $\tau$=0.5 |
| C31 | 5 | 10 | 3 continuous  2 binary | 2 continuous  2 binary | 1 continuous | continuous: 0.2 (0.5) | Uniform(-0.4,0.4) |
| C32 |  |  |  |  |  |  | Uniform(-1.0,1.0) |
| C33 | 5  (sample size from 50 to 500) | 10 | 3 continuous  2 binary | 2 continuous  2 binary | 1 continuous | continuous: 0.2 (0.5) | $\tau$=0.2 |
| C34 |  |  |  |  |  |  | $\tau$=0.5 |
| C35 | 5 | 30 | 8 continuous  7 binary | 7 continuous  7 binary | 1 continuous | continuous: 0.2 (0.5) | $\tau$=0.2 |
| C36 |  |  |  |  |  |  | $\tau$=0.5 |
| B1 | 5 | 10 | 3 continuous  2 binary | 3 continuous  2 binary | None | NA | $\tau$=0.2 |
| B2 |  |  |  |  |  |  | $\tau$=0.5 |
| B3 | 5 | 10 | 3 continuous  2 binary | 2 continuous  2 binary | 1 continuous | continuous: 0.2 (0.1) | $\tau$=0.2 |
| B4 |  |  |  |  |  |  | $\tau$=0.5 |
| B5 | 5 | 10 | 3 continuous  2 binary | 2 continuous  2 binary | 1 continuous | continuous: 0.2 (0.5) | $\tau$=0.2  $\tau$=0.5 |
| B6 |  |  |  |  |  |  |  |
| B7 | 5 | 15 | 5 continuous  3 binary | 3 continuous  2 binary | 1 continuous  1 binary | continuous: 0.2 (0.1)  binary: 0.2 (0.1) | $\tau$=0.2 |
| B8 |  |  |  |  |  |  | $\tau$=0.5 |
| B9 | 5 | 15 | 5 continuous  3 binary | 3 continuous  2 binary | 1 continuous  1 binary | continuous: 0.2 (0.5)  binary: 0.2 (0.4) | $\tau$=0.2 |
| B10 |  |  |  |  |  |  | $\tau$=0.5 |
| B11 | 5 | 15 | 5 continuous  3 binary | 2 continuous  2 binary | 2 continuous  1 binary | continuous: 0.2 (0.1)  binary: 0.2 (0.1) | $\tau$=0.2 |
| B12 |  |  |  |  |  |  | $\tau$=0.5 |
| B13 | 5 | 15 | 5 continuous  3 binary | 2 continuous  2 binary | 2 continuous  1 binary | continuous: 0.2 (0.5)  binary: 0.2 (0.4) | $\tau$=0.2 |
| B14 |  |  |  |  |  |  | $\tau$=0.5 |
| B15 | 10 | 10 | 3 continuous  2 binary | 3 continuous  2 binary | None | NA | $\tau$=0.2 |
| B16 |  |  |  |  |  |  | $\tau$=0.5 |
| B17 | 10 | 10 | 3 continuous  2 binary | 2 continuous  2 binary | 1 continuous | continuous: 0.2 (0.1) | $\tau$=0.2 |
| B18 |  |  |  |  |  |  | $\tau$=0.5 |
| B19 | 10 | 10 | 3 continuous  2 binary | 2 continuous  2 binary | 1 continuous | continuous: 0.2 (0.5) | $\tau$=0.2 |
| B20 |  |  |  |  |  |  | $\tau$=0.5 |
| B21 | 10 | 15 | 5 continuous  3 binary | 3 continuous  2 binary | 1 continuous  1 binary | continuous: 0.2 (0.1)  binary: 0.2 (0.1) | $\tau$=0.2 |
| B22 |  |  |  |  |  |  | $\tau$=0.5 |
| B23 | 10 | 15 | 5 continuous  3 binary | 3 continuous  2 binary | 1 continuous  1 binary | continuous: 0.2 (0.5)  binary: 0.2 (0.4) | $\tau$=0.2 |
| B24 |  |  |  |  |  |  | $\tau$=0.5 |
| B25 | 10 | 15 | 5 continuous  3 binary | 2 continuous  2 binary | 2 continuous  1 binary | continuous: 0.2 (0.1)  binary: 0.2 (0.1) | $\tau$=0.2 |
| B26 |  |  |  |  |  |  | $\tau$=0.5 |
| B27 | 10 | 15 | 5 continuous  3 binary | 2 continuous  2 binary | 2 continuous  1 binary | continuous: 0.2 (0.5)  binary: 0.2 (0.4) | $\tau$=0.2 |
| B28 |  |  |  |  |  |  | $\tau$=0.5 |
| B29 | 5 | 10 | None | None | 5 continuous  5 binary | continuous: 0.2 (0.5)  binary: 0.2 (0.4) | $\tau$=0.2 |
| B30 |  |  |  |  |  |  | $\tau$=0.5 |
| B31 | 5 | 10 | 3 continuous  2 binary | 2 continuous  2 binary | 1 continuous | continuous: 0.2 (0.1) | Uniform(-0.4,0.4) |
| B32 |  |  |  |  |  |  | Uniform(-1.0,1.0) |
| B33 | 5  (sample size from 50 to 500) | 10 | 3 continuous  2 binary | 2 continuous  2 binary | 1 continuous | continuous: 0.2 (0.1) | $\tau$=0.2 |
| B34 |  |  |  |  |  |  | $\tau$=0.5 |
| B35 | 5 | 30 | 8 continuous  7 binary | 7 continuous  7 binary | 1 continuous | continuous: 0.2 (0.1) | $\tau$=0.2 |
| B36 |  |  |  |  |  |  | $\tau$=0.5 |

# Results from simulations

**Figure 1:** Results from simulations of a continuous outcome, comparing different methods in terms of the mean squared error (MSE) for the patient-specific treatment effect grouped by heterogeneity of the treatment effect. Scenarios C1-C36 are shown in pairs, differing only in the assumed heterogeneity ($\tau$). Within each pair, the two scenarios explore the same type of outcome, have the same number of covariates and effect modifiers, equal heterogeneity and the number of studies. Scenarios are described in detail in Section 2 of the Appendix. A: GLMM-oracle; B: GLMM-full; C: STEP; D: LASSO; E: ridge; F: adaptive LASSO; G: Bayesian LASSO; H: SSVS.

***
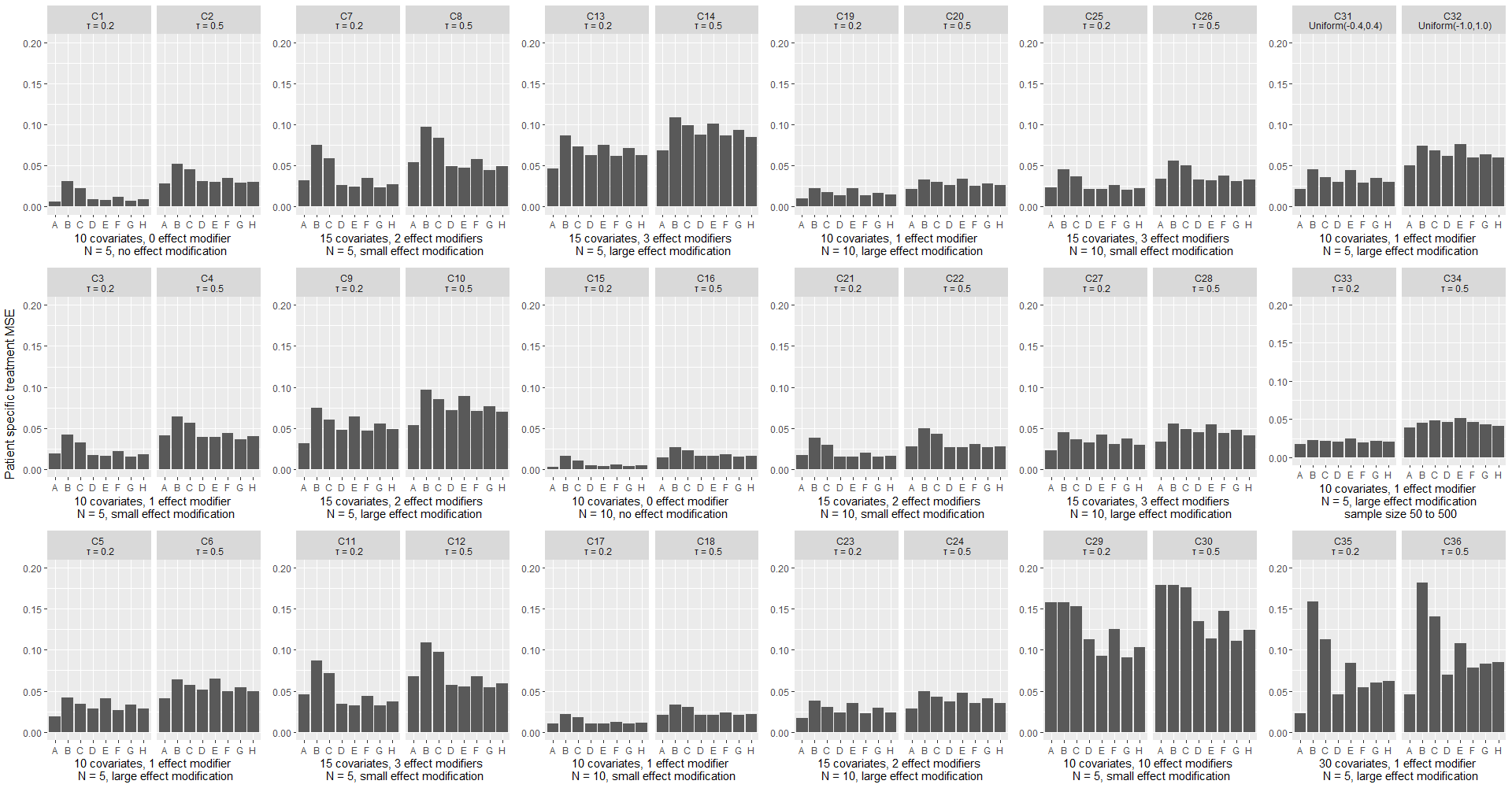
***

**Figure 2:** Results from simulations of a binary outcome, comparing different methods in terms of the mean squared error (MSE) for the patient-specific treatment effect grouped by heterogeneity of the treatment effect. Scenarios B1-B36 are shown in pairs, differing only in the assumed heterogeneity ($\tau$). Within each pair, the two scenarios explore the same type of outcome, have the same number of covariates and effect modifiers, equal heterogeneity and the number of studies. Scenarios are described in detail in Section 2 of the Appendix. A: GLMM-oracle; B: GLMM-full; C: STEP; D: LASSO; E: ridge; F: adaptive LASSO; G: Bayesian LASSO; H: SSVS.

***
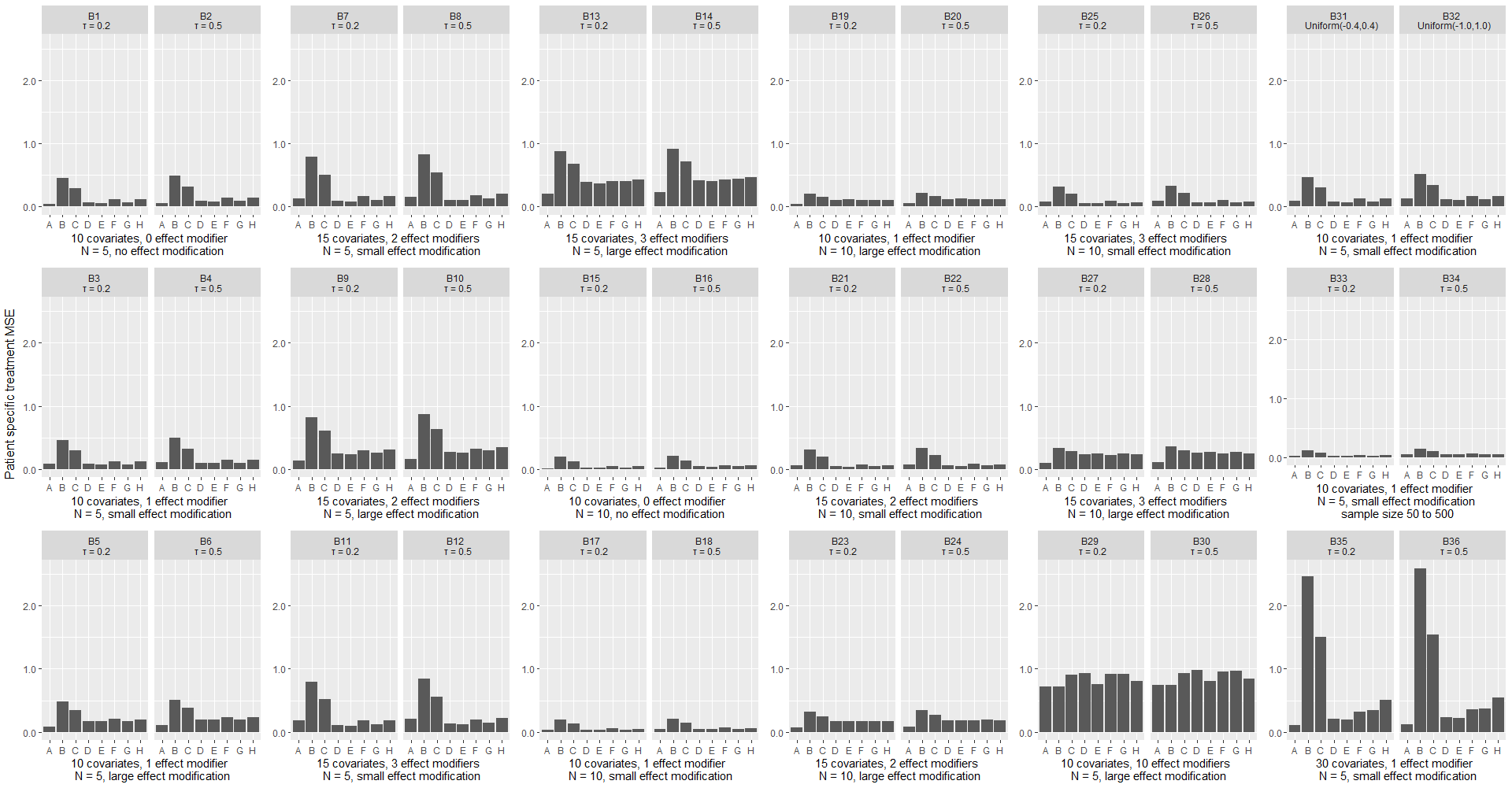
***

**Figure 3**: Simulation results of a continuous outcome comparing different methods in terms of true effect modifiers MSE. A: GLMM-oracle; B: GLMM-full; C: STEP; D: LASSO; E: ridge; F: adaptive LASSO; G: Bayesian LASSO; H: SSVS.


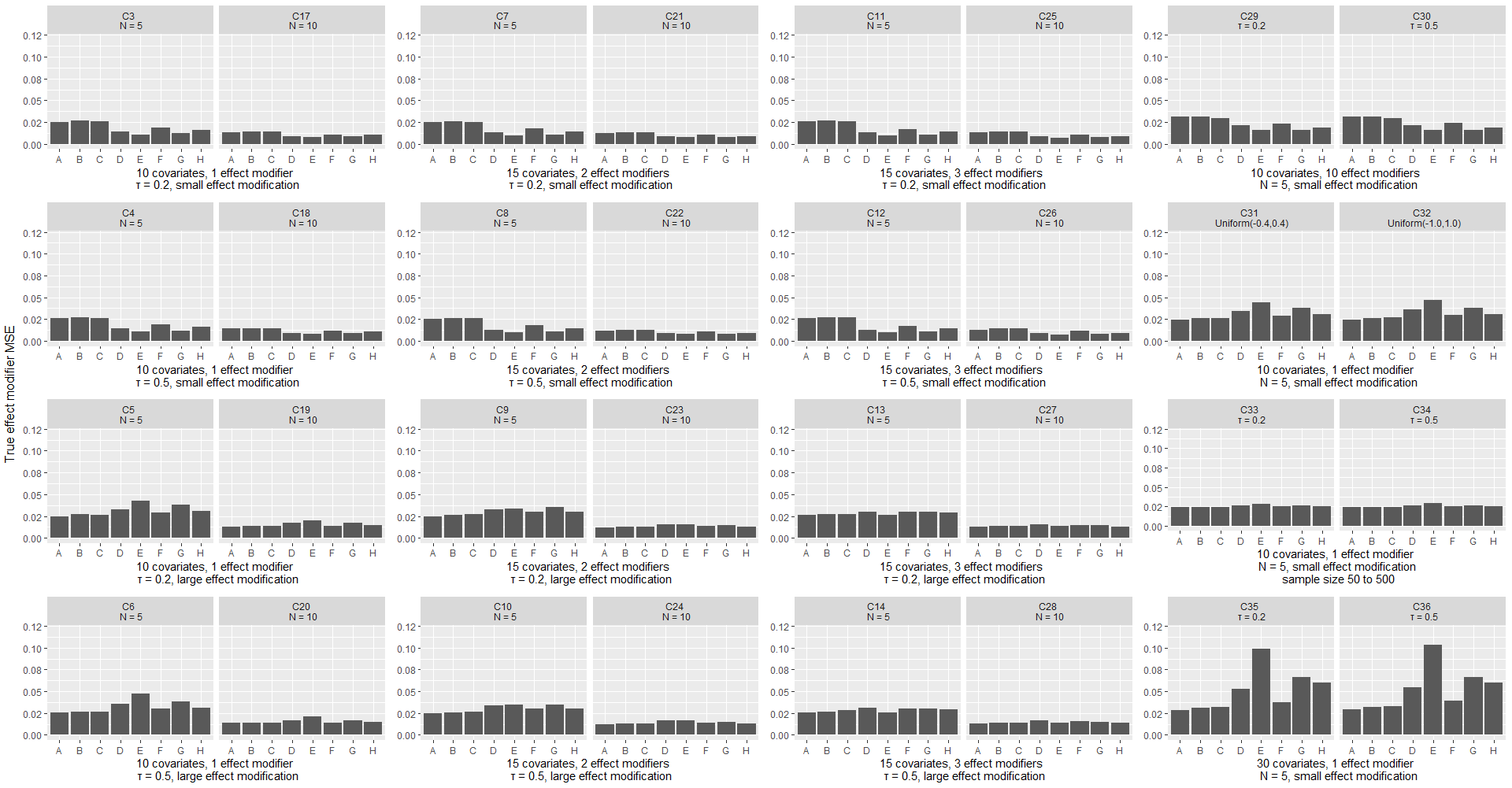


**Figure 4**: Simulation results of a binary outcome comparing different methods in terms of true effect modifiers MSE. A: GLMM-oracle; B: GLMM-full; C: STEP; D: LASSO; E: ridge; F: adaptive LASSO; G: Bayesian LASSO; H: SSVS.


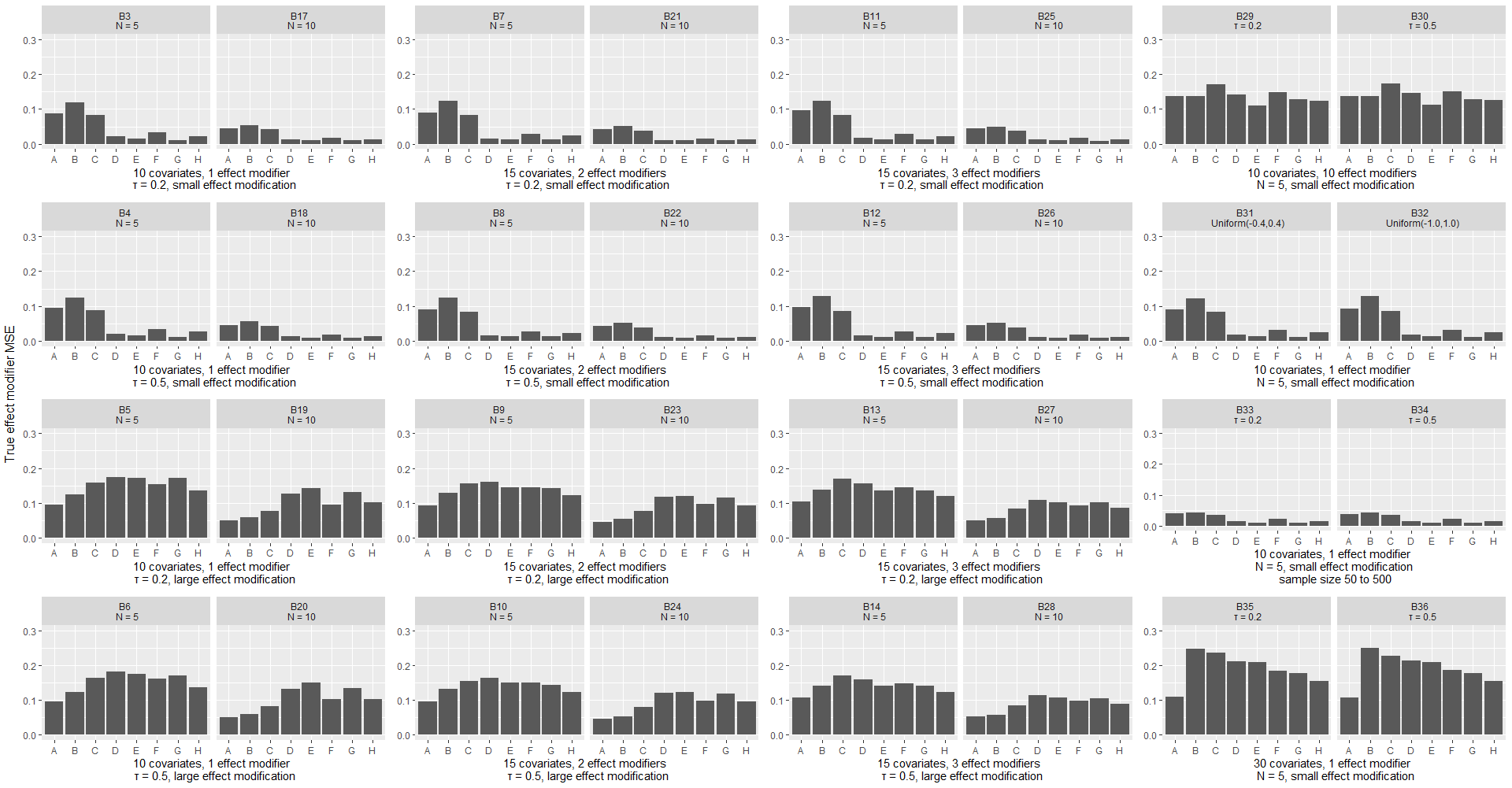


**Figure 5:** Simulation results of a continuous outcome comparing different methods in terms of false effect modifier MSE. A: GLMM-oracle; B: GLMM-full; C: STEP; D: LASSO; E: ridge; F: adaptive LASSO; G: Bayesian LASSO; H: SSVS.


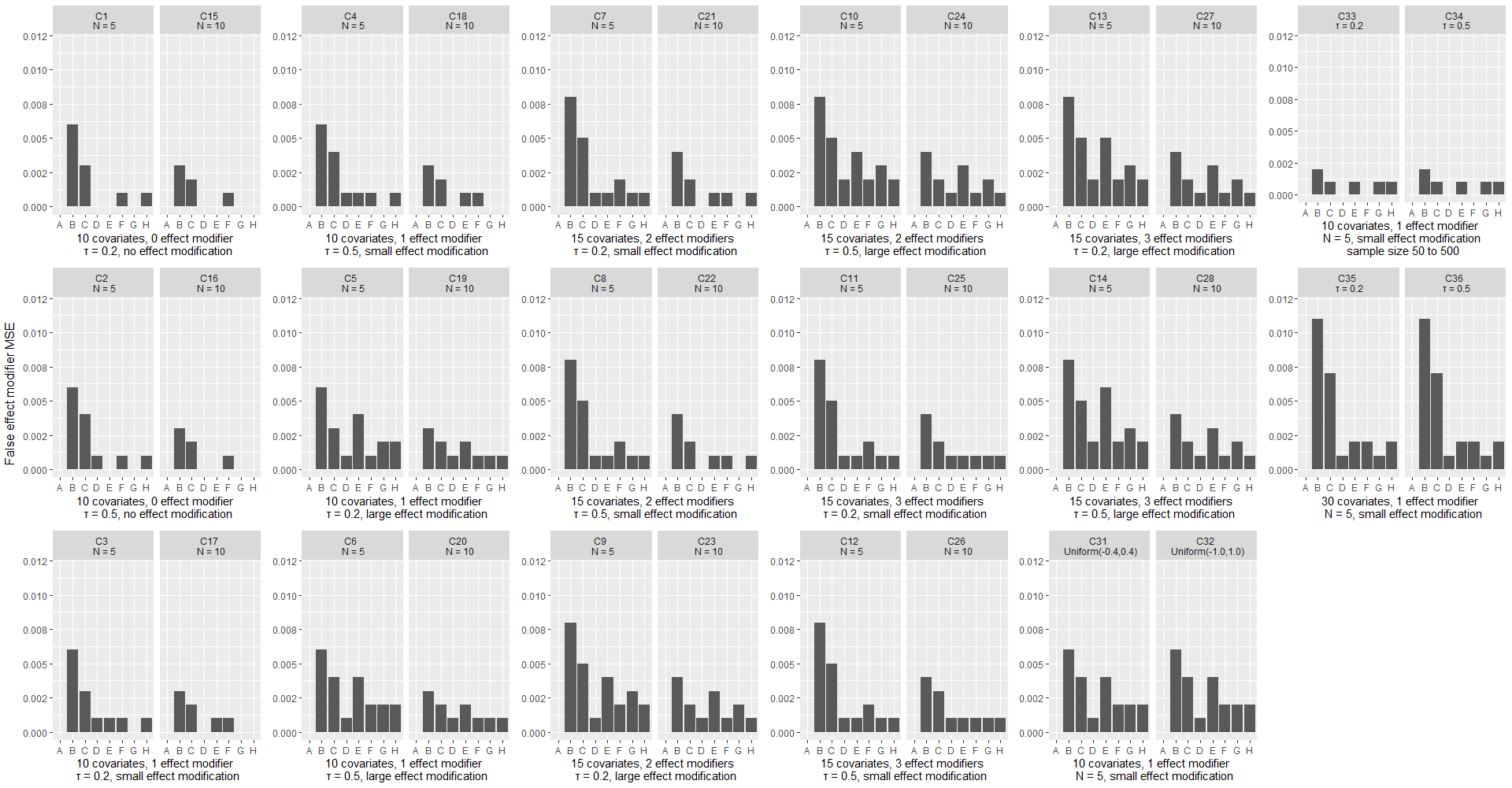


**Figure 6:** Simulation results of a binary outcome comparing different methods in terms of false effect modifier MSE. A: GLMM-oracle; B: GLMM-full; C: STEP; D: LASSO; E: ridge; F: adaptive LASSO; G: Bayesian LASSO; H: SSVS.


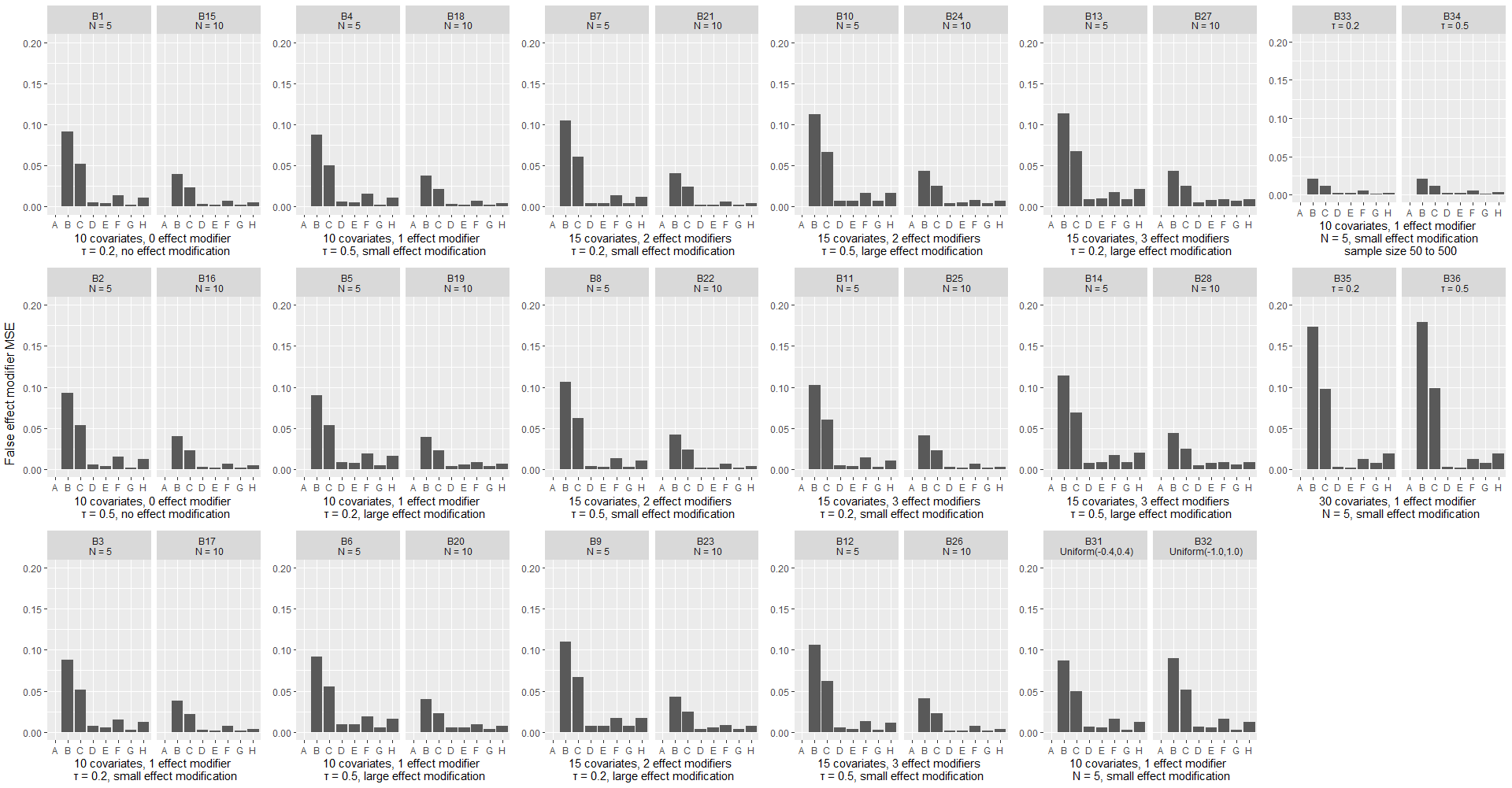


**Table** **4:** Full results from the simulations. Model abbreviations as per Section 2 in the main paper. MSE: mean squared error. SE: standard error. To facilitate readers, the lowest patient-specific treatment-effect MSE is shown in bold (excluding MSE of GLMM-oracle)

| **Scenario** | **Model** | **False effect modifier MSE** | **True effect modifier MSE** | **Treatment MSE** | **Patient specific treatment effect MSE** | **Continuous effect modifier SE** | **Binary effect modifier SE** | **Treatment effect SE** |
| --- | --- | --- | --- | --- | --- | --- | --- | --- |
| C1 | GLMM-oracle | 0 |  | 0.012 | 0.006 |  |  | 0.109 |
|  | GLMM-full | 0.006 |  | 0.013 | 0.031 |  |  | 0.109 |
|  | STEP | 0.003 |  | 0.013 | 0.022 |  |  | 0.068 |
|  | LASSO | 0 |  | 0.013 | 0.009 |  |  |  |
|  | ridge | 0 |  | 0.013 | 0.008 |  |  |  |
|  | adaptive LASSO | 0.001 |  | 0.013 | 0.012 |  |  |  |
|  | Bayesian LASSO | 0 |  | 0.012 | **0.007** |  |  | 0.167 |
|  | SSVS | 0.001 |  | 0.013 | 0.009 |  |  | 0.167 |
| C2 | GLMM-oracle | 0 |  | 0.055 | 0.028 |  |  | 0.223 |
|  | GLMM-full | 0.006 |  | 0.055 | 0.052 |  |  | 0.223 |
|  | STEP | 0.004 |  | 0.057 | 0.045 |  |  | 0.071 |
|  | LASSO | 0.001 |  | 0.057 | 0.031 |  |  |  |
|  | ridge | 0 |  | 0.057 | 0.030 |  |  |  |
|  | adaptive LASSO | 0.001 |  | 0.057 | 0.035 |  |  |  |
|  | Bayesian LASSO | 0 |  | 0.055 | **0.029** |  |  | 0.305 |
|  | SSVS | 0.001 |  | 0.055 | 0.030 |  |  | 0.305 |
| C3 | GLMM-oracle | 0 | 0.025 | 0.013 | 0.019 | 0.070 |  | 0.111 |
|  | GLMM-full | 0.006 | 0.027 | 0.013 | 0.042 | 0.078 |  | 0.111 |
|  | STEP | 0.003 | 0.026 | 0.013 | 0.033 | 0.072 |  | 0.070 |
|  | LASSO | 0.001 | 0.014 | 0.013 | 0.017 |  |  |  |
|  | ridge | 0.001 | 0.011 | 0.013 | 0.016 |  |  |  |
|  | adaptive LASSO | 0.001 | 0.019 | 0.013 | 0.022 |  |  |  |
|  | Bayesian LASSO | 0 | 0.012 | 0.013 | **0.015** | 0.050 |  | 0.169 |
|  | SSVS | 0.001 | 0.016 | 0.013 | 0.018 | 0.059 |  | 0.169 |
| C4 | GLMM-oracle | 0 | 0.026 | 0.055 | 0.041 | 0.070 |  | 0.224 |
|  | GLMM-full | 0.006 | 0.027 | 0.056 | 0.064 | 0.078 |  | 0.225 |
|  | STEP | 0.004 | 0.026 | 0.058 | 0.057 | 0.075 |  | 0.074 |
|  | LASSO | 0.001 | 0.014 | 0.057 | 0.039 |  |  |  |
|  | ridge | 0.001 | 0.011 | 0.057 | 0.039 |  |  |  |
|  | adaptive LASSO | 0.001 | 0.019 | 0.057 | 0.044 |  |  |  |
|  | Bayesian LASSO | 0 | 0.012 | 0.055 | **0.036** | 0.051 |  | 0.307 |
|  | SSVS | 0.001 | 0.016 | 0.055 | 0.040 | 0.059 |  | 0.306 |
| C5 | GLMM-oracle | 0 | 0.025 | 0.013 | 0.019 | 0.070 |  | 0.111 |
|  | GLMM-full | 0.006 | 0.027 | 0.013 | 0.042 | 0.078 |  | 0.111 |
|  | STEP | 0.003 | 0.026 | 0.013 | 0.034 | 0.072 |  | 0.070 |
|  | LASSO | 0.001 | 0.033 | 0.013 | 0.028 |  |  |  |
|  | ridge | 0.004 | 0.043 | 0.013 | 0.041 |  |  |  |
|  | adaptive LASSO | 0.001 | 0.029 | 0.013 | **0.027** |  |  |  |
|  | Bayesian LASSO | 0.002 | 0.038 | 0.013 | 0.033 | 0.079 |  | 0.169 |
|  | SSVS | 0.002 | 0.031 | 0.013 | 0.028 | 0.077 |  | 0.170 |
| C6 | GLMM-oracle | 0 | 0.026 | 0.055 | 0.041 | 0.070 |  | 0.224 |
|  | GLMM-full | 0.006 | 0.027 | 0.056 | 0.064 | 0.078 |  | 0.225 |
|  | STEP | 0.004 | 0.027 | 0.058 | 0.057 | 0.075 |  | 0.074 |
|  | LASSO | 0.001 | 0.036 | 0.057 | 0.051 |  |  |  |
|  | ridge | 0.004 | 0.047 | 0.057 | 0.065 |  |  |  |
|  | adaptive LASSO | 0.002 | 0.03 | 0.057 | **0.050** |  |  |  |
|  | Bayesian LASSO | 0.002 | 0.038 | 0.055 | 0.054 | 0.079 |  | 0.306 |
|  | SSVS | 0.002 | 0.031 | 0.055 | **0.050** | 0.077 |  | 0.306 |
| C7 | GLMM-oracle | 0 | 0.025 | 0.015 | 0.032 | 0.078 | 0.078 | 0.116 |
|  | GLMM-full | 0.008 | 0.026 | 0.015 | 0.075 | 0.088 | 0.080 | 0.118 |
|  | STEP | 0.005 | 0.025 | 0.015 | 0.059 | 0.080 | 0.078 | 0.078 |
|  | LASSO | 0.001 | 0.013 | 0.015 | 0.026 |  |  |  |
|  | ridge | 0.001 | 0.010 | 0.015 | 0.024 |  |  |  |
|  | adaptive LASSO | 0.002 | 0.018 | 0.015 | 0.035 |  |  |  |
|  | Bayesian LASSO | 0.001 | 0.011 | 0.015 | **0.023** | 0.054 | 0.053 | 0.176 |
|  | SSVS | 0.001 | 0.014 | 0.015 | 0.027 | 0.061 | 0.060 | 0.176 |
| C8 | GLMM-oracle | 0 | 0.025 | 0.057 | 0.054 | 0.078 | 0.078 | 0.227 |
|  | GLMM-full | 0.008 | 0.026 | 0.057 | 0.097 | 0.088 | 0.080 | 0.228 |
|  | STEP | 0.005 | 0.026 | 0.059 | 0.084 | 0.083 | 0.082 | 0.081 |
|  | LASSO | 0.001 | 0.013 | 0.059 | 0.049 |  |  |  |
|  | ridge | 0.001 | 0.010 | 0.059 | 0.047 |  |  |  |
|  | adaptive LASSO | 0.002 | 0.018 | 0.059 | 0.058 |  |  |  |
|  | Bayesian LASSO | 0.001 | 0.011 | 0.057 | **0.044** | 0.054 | 0.053 | 0.307 |
|  | SSVS | 0.001 | 0.014 | 0.057 | 0.049 | 0.061 | 0.060 | 0.308 |
| C9 | GLMM-oracle | 0 | 0.025 | 0.015 | 0.032 | 0.078 | 0.078 | 0.116 |
|  | GLMM-full | 0.008 | 0.026 | 0.015 | 0.075 | 0.088 | 0.080 | 0.118 |
|  | STEP | 0.005 | 0.027 | 0.015 | 0.060 | 0.081 | 0.079 | 0.078 |
|  | LASSO | 0.001 | 0.033 | 0.015 | 0.048 |  |  |  |
|  | ridge | 0.004 | 0.034 | 0.015 | 0.064 |  |  |  |
|  | adaptive LASSO | 0.002 | 0.03 | 0.01 | **0.047** |  |  |  |
|  | Bayesian LASSO | 0.003 | 0.035 | 0.015 | 0.056 | 0.087 | 0.080 | 0.177 |
|  | SSVS | 0.002 | 0.030 | 0.015 | 0.049 | 0.086 | 0.080 | 0.177 |
| C10 | GLMM-oracle | 0 | 0.025 | 0.057 | 0.054 | 0.078 | 0.078 | 0.227 |
|  | GLMM-full | 0.008 | 0.026 | 0.057 | 0.097 | 0.088 | 0.080 | 0.228 |
|  | STEP | 0.005 | 0.027 | 0.059 | 0.085 | 0.084 | 0.082 | 0.081 |
|  | LASSO | 0.002 | 0.034 | 0.059 | 0.072 |  |  |  |
|  | ridge | 0.004 | 0.035 | 0.059 | 0.089 |  |  |  |
|  | adaptive LASSO | 0.002 | 0.03 | 0.059 | 0.071 |  |  |  |
|  | Bayesian LASSO | 0.003 | 0.035 | 0.057 | 0.077 | 0.087 | 0.081 | 0.308 |
|  | SSVS | 0.002 | 0.030 | 0.057 | **0.070** | 0.086 | 0.081 | 0.308 |
| C11 | GLMM-oracle | 0 | 0.026 | 0.016 | 0.046 | 0.085 | 0.080 | 0.118 |
|  | GLMM-full | 0.008 | 0.027 | 0.016 | 0.087 | 0.091 | 0.083 | 0.118 |
|  | STEP | 0.005 | 0.026 | 0.016 | 0.072 | 0.084 | 0.081 | 0.081 |
|  | LASSO | 0.001 | 0.013 | 0.016 | 0.034 |  |  |  |
|  | ridge | 0.001 | 0.010 | 0.016 | **0.032** |  |  |  |
|  | adaptive LASSO | 0.002 | 0.017 | 0.016 | 0.044 |  |  |  |
|  | Bayesian LASSO | 0.001 | 0.011 | 0.016 | **0.032** | 0.059 | 0.057 | 0.178 |
|  | SSVS | 0.001 | 0.014 | 0.016 | 0.037 | 0.066 | 0.063 | 0.177 |
| C12 | GLMM-oracle | 0 | 0.026 | 0.059 | 0.068 | 0.085 | 0.081 | 0.227 |
|  | GLMM-full | 0.008 | 0.027 | 0.060 | 0.109 | 0.091 | 0.083 | 0.227 |
|  | STEP | 0.005 | 0.027 | 0.062 | 0.097 | 0.087 | 0.083 | 0.084 |
|  | LASSO | 0.001 | 0.013 | 0.061 | 0.057 |  |  |  |
|  | ridge | 0.001 | 0.010 | 0.061 | 0.055 |  |  |  |
|  | adaptive LASSO | 0.002 | 0.017 | 0.061 | 0.068 |  |  |  |
|  | Bayesian LASSO | 0.001 | 0.011 | 0.060 | **0.054** | 0.059 | 0.057 | 0.307 |
|  | SSVS | 0.001 | 0.014 | 0.060 | 0.059 | 0.066 | 0.063 | 0.307 |
| C13 | GLMM-oracle | 0 | 0.026 | 0.016 | 0.046 | 0.085 | 0.080 | 0.118 |
|  | GLMM-full | 0.008 | 0.027 | 0.016 | 0.087 | 0.091 | 0.083 | 0.118 |
|  | STEP | 0.005 | 0.027 | 0.016 | 0.073 | 0.086 | 0.081 | 0.081 |
|  | LASSO | 0.002 | 0.030 | 0.016 | 0.063 |  |  |  |
|  | ridge | 0.005 | 0.026 | 0.016 | 0.075 |  |  |  |
|  | adaptive LASSO | 0.002 | 0.03 | 0.016 | **0.062** |  |  |  |
|  | Bayesian LASSO | 0.003 | 0.030 | 0.016 | 0.071 | 0.090 | 0.083 | 0.178 |
|  | SSVS | 0.002 | 0.029 | 0.016 | 0.063 | 0.089 | 0.083 | 0.178 |
| C14 | GLMM-oracle | 0 | 0.026 | 0.059 | 0.068 | 0.085 | 0.081 | 0.227 |
|  | GLMM-full | 0.008 | 0.027 | 0.060 | 0.109 | 0.091 | 0.083 | 0.227 |
|  | STEP | 0.005 | 0.028 | 0.062 | 0.099 | 0.089 | 0.084 | 0.084 |
|  | LASSO | 0.002 | 0.031 | 0.061 | 0.088 |  |  |  |
|  | ridge | 0.006 | 0.026 | 0.061 | 0.101 |  |  |  |
|  | adaptive LASSO | 0.002 | 0.03 | 0.061 | 0.087 |  |  |  |
|  | Bayesian LASSO | 0.003 | 0.030 | 0.060 | 0.093 | 0.090 | 0.083 | 0.307 |
|  | SSVS | 0.002 | 0.029 | 0.060 | **0.085** | 0.089 | 0.084 | 0.307 |
| C15 | GLMM-oracle | 0 |  | 0.006 | 0.003 |  |  | 0.078 |
|  | GLMM-full | 0.003 |  | 0.006 | 0.016 |  |  | 0.078 |
|  | STEP | 0.002 |  | 0.006 | 0.011 |  |  | 0.049 |
|  | LASSO | 0 |  | 0.007 | 0.005 |  |  |  |
|  | ridge | 0 |  | 0.007 | **0.004** |  |  |  |
|  | adaptive LASSO | 0.001 |  | 0.007 | 0.006 |  |  |  |
|  | Bayesian LASSO | 0 |  | 0.006 | **0.004** |  |  | 0.092 |
|  | SSVS | 0 |  | 0.006 | 0.005 |  |  | 0.092 |
| C16 | GLMM-oracle | 0 |  | 0.028 | 0.014 |  |  | 0.161 |
|  | GLMM-full | 0.003 |  | 0.028 | 0.027 |  |  | 0.161 |
|  | STEP | 0.002 |  | 0.029 | 0.023 |  |  | 0.051 |
|  | LASSO | 0 |  | 0.030 | 0.016 |  |  |  |
|  | ridge | 0 |  | 0.030 | 0.016 |  |  |  |
|  | adaptive LASSO | 0.001 |  | 0.03 | 0.018 |  |  |  |
|  | Bayesian LASSO | 0 |  | 0.028 | **0.015** |  |  | 0.187 |
|  | SSVS | 0 |  | 0.028 | 0.016 |  |  | 0.187 |
| C17 | GLMM-oracle | 0 | 0.013 | 0.007 | 0.010 | 0.051 |  | 0.079 |
|  | GLMM-full | 0.003 | 0.014 | 0.007 | 0.022 | 0.056 |  | 0.080 |
|  | STEP | 0.002 | 0.014 | 0.007 | 0.018 | 0.052 |  | 0.051 |
|  | LASSO | 0 | 0.009 | 0.007 | **0.010** |  |  |  |
|  | ridge | 0.001 | 0.008 | 0.007 | **0.010** |  |  |  |
|  | adaptive LASSO | 0.001 | 0.011 | 0.007 | 0.012 |  |  |  |
|  | Bayesian LASSO | 0 | 0.009 | 0.007 | **0.010** | 0.041 |  | 0.093 |
|  | SSVS | 0 | 0.011 | 0.007 | 0.011 | 0.045 |  | 0.093 |
| C18 | GLMM-oracle | 0 | 0.014 | 0.028 | 0.021 | 0.051 |  | 0.161 |
|  | GLMM-full | 0.003 | 0.014 | 0.028 | 0.033 | 0.056 |  | 0.161 |
|  | STEP | 0.002 | 0.014 | 0.029 | 0.030 | 0.054 |  | 0.053 |
|  | LASSO | 0 | 0.009 | 0.029 | **0.021** |  |  |  |
|  | ridge | 0.001 | 0.008 | 0.029 | **0.021** |  |  |  |
|  | adaptive LASSO | 0.001 | 0.012 | 0.029 | 0.024 |  |  |  |
|  | Bayesian LASSO | 0 | 0.009 | 0.028 | **0.021** | 0.041 |  | 0.188 |
|  | SSVS | 0 | 0.011 | 0.028 | 0.022 | 0.045 |  | 0.188 |
| C19 | GLMM-oracle | 0 | 0.013 | 0.007 | 0.010 | 0.051 |  | 0.079 |
|  | GLMM-full | 0.003 | 0.014 | 0.007 | 0.022 | 0.056 |  | 0.080 |
|  | STEP | 0.002 | 0.014 | 0.007 | 0.018 | 0.052 |  | 0.051 |
|  | LASSO | 0.001 | 0.017 | 0.007 | **0.014** |  |  |  |
|  | ridge | 0.002 | 0.020 | 0.007 | 0.022 |  |  |  |
|  | adaptive LASSO | 0.001 | 0.014 | 0.007 | **0.014** |  |  |  |
|  | Bayesian LASSO | 0.001 | 0.017 | 0.007 | 0.017 | 0.056 |  | 0.093 |
|  | SSVS | 0.001 | 0.015 | 0.007 | 0.015 | 0.055 |  | 0.093 |
| C20 | GLMM-oracle | 0 | 0.014 | 0.028 | 0.021 | 0.051 |  | 0.161 |
|  | GLMM-full | 0.003 | 0.014 | 0.028 | 0.033 | 0.056 |  | 0.161 |
|  | STEP | 0.002 | 0.014 | 0.029 | 0.030 | 0.055 |  | 0.053 |
|  | LASSO | 0.001 | 0.017 | 0.029 | 0.026 |  |  |  |
|  | ridge | 0.002 | 0.021 | 0.029 | 0.034 |  |  |  |
|  | adaptive LASSO | 0.001 | 0.014 | 0.029 | **0.025** |  |  |  |
|  | Bayesian LASSO | 0.001 | 0.017 | 0.028 | 0.028 | 0.056 |  | 0.188 |
|  | SSVS | 0.001 | 0.015 | 0.028 | 0.026 | 0.055 |  | 0.188 |
| C21 | GLMM-oracle | 0 | 0.012 | 0.008 | 0.017 | 0.057 | 0.056 | 0.083 |
|  | GLMM-full | 0.004 | 0.013 | 0.008 | 0.038 | 0.063 | 0.057 | 0.083 |
|  | STEP | 0.002 | 0.013 | 0.008 | 0.030 | 0.058 | 0.057 | 0.057 |
|  | LASSO | 0 | 0.009 | 0.007 | **0.015** |  |  |  |
|  | ridge | 0.001 | 0.008 | 0.007 | **0.015** |  |  |  |
|  | adaptive LASSO | 0.001 | 0.011 | 0.007 | 0.020 |  |  |  |
|  | Bayesian LASSO | 0 | 0.008 | 0.008 | **0.015** | 0.044 | 0.043 | 0.097 |
|  | SSVS | 0.001 | 0.009 | 0.008 | 0.016 | 0.047 | 0.046 | 0.097 |
| C22 | GLMM-oracle | 0 | 0.012 | 0.031 | 0.028 | 0.057 | 0.057 | 0.163 |
|  | GLMM-full | 0.004 | 0.013 | 0.031 | 0.050 | 0.063 | 0.057 | 0.163 |
|  | STEP | 0.002 | 0.013 | 0.032 | 0.043 | 0.060 | 0.059 | 0.059 |
|  | LASSO | 0 | 0.009 | 0.030 | **0.027** |  |  |  |
|  | ridge | 0.001 | 0.008 | 0.030 | **0.027** |  |  |  |
|  | adaptive LASSO | 0.001 | 0.011 | 0.03 | 0.031 |  |  |  |
|  | Bayesian LASSO | 0 | 0.008 | 0.031 | **0.027** | 0.044 | 0.043 | 0.189 |
|  | SSVS | 0.001 | 0.009 | 0.031 | 0.028 | 0.047 | 0.046 | 0.189 |
| C23 | GLMM-oracle | 0 | 0.012 | 0.008 | 0.017 | 0.057 | 0.056 | 0.083 |
|  | GLMM-full | 0.004 | 0.013 | 0.008 | 0.038 | 0.063 | 0.057 | 0.083 |
|  | STEP | 0.002 | 0.013 | 0.008 | 0.030 | 0.058 | 0.057 | 0.057 |
|  | LASSO | 0.001 | 0.016 | 0.007 | 0.024 |  |  |  |
|  | ridge | 0.003 | 0.016 | 0.007 | 0.035 |  |  |  |
|  | adaptive LASSO | 0.001 | 0.014 | 0.007 | **0.023** |  |  |  |
|  | Bayesian LASSO | 0.002 | 0.015 | 0.008 | 0.029 | 0.062 | 0.058 | 0.097 |
|  | SSVS | 0.001 | 0.013 | 0.008 | 0.024 | 0.061 | 0.057 | 0.097 |
| C24 | GLMM-oracle | 0 | 0.012 | 0.031 | 0.028 | 0.057 | 0.057 | 0.163 |
|  | GLMM-full | 0.004 | 0.013 | 0.031 | 0.050 | 0.063 | 0.057 | 0.163 |
|  | STEP | 0.002 | 0.013 | 0.032 | 0.043 | 0.060 | 0.059 | 0.059 |
|  | LASSO | 0.001 | 0.017 | 0.030 | 0.037 |  |  |  |
|  | ridge | 0.003 | 0.017 | 0.030 | 0.048 |  |  |  |
|  | adaptive LASSO | 0.001 | 0.014 | 0.03 | **0.035** |  |  |  |
|  | Bayesian LASSO | 0.002 | 0.015 | 0.031 | 0.041 | 0.062 | 0.058 | 0.189 |
|  | SSVS | 0.001 | 0.013 | 0.031 | **0.035** | 0.061 | 0.057 | 0.189 |
| C25 | GLMM-oracle | 0 | 0.013 | 0.008 | 0.023 | 0.062 | 0.059 | 0.084 |
|  | GLMM-full | 0.004 | 0.014 | 0.008 | 0.045 | 0.065 | 0.059 | 0.085 |
|  | STEP | 0.002 | 0.014 | 0.008 | 0.037 | 0.061 | 0.059 | 0.059 |
|  | LASSO | 0.001 | 0.009 | 0.008 | 0.021 |  |  |  |
|  | ridge | 0.001 | 0.007 | 0.008 | 0.021 |  |  |  |
|  | adaptive LASSO | 0.001 | 0.011 | 0.008 | 0.026 |  |  |  |
|  | Bayesian LASSO | 0.001 | 0.008 | 0.008 | **0.020** | 0.048 | 0.047 | 0.099 |
|  | SSVS | 0.001 | 0.009 | 0.008 | 0.022 | 0.051 | 0.049 | 0.099 |
| C26 | GLMM-oracle | 0 | 0.013 | 0.030 | 0.034 | 0.062 | 0.059 | 0.164 |
|  | GLMM-full | 0.004 | 0.014 | 0.030 | 0.056 | 0.065 | 0.060 | 0.164 |
|  | STEP | 0.003 | 0.014 | 0.031 | 0.050 | 0.063 | 0.061 | 0.061 |
|  | LASSO | 0.001 | 0.009 | 0.030 | 0.033 |  |  |  |
|  | ridge | 0.001 | 0.007 | 0.030 | 0.032 |  |  |  |
|  | adaptive LASSO | 0.001 | 0.012 | 0.030 | 0.038 |  |  |  |
|  | Bayesian LASSO | 0.001 | 0.008 | 0.030 | **0.031** | 0.048 | 0.047 | 0.190 |
|  | SSVS | 0.001 | 0.009 | 0.030 | 0.033 | 0.051 | 0.049 | 0.190 |
| C27 | GLMM-oracle | 0 | 0.013 | 0.008 | 0.023 | 0.062 | 0.059 | 0.084 |
|  | GLMM-full | 0.004 | 0.014 | 0.008 | 0.045 | 0.065 | 0.059 | 0.085 |
|  | STEP | 0.002 | 0.014 | 0.008 | 0.036 | 0.063 | 0.059 | 0.059 |
|  | LASSO | 0.001 | 0.016 | 0.008 | 0.033 |  |  |  |
|  | ridge | 0.003 | 0.014 | 0.008 | 0.042 |  |  |  |
|  | adaptive LASSO | 0.001 | 0.015 | 0.008 | 0.031 |  |  |  |
|  | Bayesian LASSO | 0.002 | 0.015 | 0.008 | 0.037 | 0.065 | 0.060 | 0.099 |
|  | SSVS | 0.001 | 0.013 | 0.008 | **0.030** | 0.063 | 0.060 | 0.099 |
| C28 | GLMM-oracle | 0 | 0.013 | 0.030 | 0.034 | 0.062 | 0.059 | 0.164 |
|  | GLMM-full | 0.004 | 0.014 | 0.030 | 0.056 | 0.065 | 0.060 | 0.164 |
|  | STEP | 0.002 | 0.014 | 0.031 | 0.049 | 0.065 | 0.061 | 0.061 |
|  | LASSO | 0.001 | 0.017 | 0.030 | 0.045 |  |  |  |
|  | ridge | 0.003 | 0.014 | 0.030 | 0.055 |  |  |  |
|  | adaptive LASSO | 0.001 | 0.016 | 0.03 | 0.044 |  |  |  |
|  | Bayesian LASSO | 0.002 | 0.015 | 0.030 | 0.048 | 0.065 | 0.060 | 0.190 |
|  | SSVS | 0.001 | 0.014 | 0.030 | **0.041** | 0.063 | 0.060 | 0.190 |
| C29 | GLMM-oracle |  | 0.031 | 0.019 | 0.158 | 0.111 | 0.103 | 0.135 |
|  | GLMM-full |  | 0.031 | 0.019 | 0.158 | 0.111 | 0.103 | 0.135 |
|  | STEP |  | 0.030 | 0.019 | 0.153 | 0.105 | 0.102 | 0.101 |
|  | LASSO |  | 0.021 | 0.018 | 0.113 |  |  |  |
|  | ridge |  | 0.016 | 0.018 | 0.093 |  |  |  |
|  | adaptive LASSO |  | 0.023 | 0.018 | 0.125 |  |  |  |
|  | Bayesian LASSO |  | 0.016 | 0.019 | **0.091** | 0.087 | 0.083 | 0.199 |
|  | SSVS |  | 0.019 | 0.019 | 0.103 | 0.090 | 0.085 | 0.199 |
| C30 | GLMM-oracle |  | 0.031 | 0.061 | 0.179 | 0.111 | 0.103 | 0.235 |
|  | GLMM-full |  | 0.031 | 0.061 | 0.179 | 0.111 | 0.103 | 0.235 |
|  | STEP |  | 0.030 | 0.062 | 0.176 | 0.107 | 0.104 | 0.104 |
|  | LASSO |  | 0.021 | 0.062 | 0.135 |  |  |  |
|  | ridge |  | 0.016 | 0.062 | 0.114 |  |  |  |
|  | adaptive LASSO |  | 0.024 | 0.062 | 0.147 |  |  |  |
|  | Bayesian LASSO |  | 0.016 | 0.061 | **0.111** | 0.087 | 0.083 | 0.314 |
|  | SSVS |  | 0.019 | 0.061 | 0.124 | 0.090 | 0.085 | 0.314 |
| C31 | GLMM-oracle | 0.000 | 0.024 | 0.017 | 0.021 | 0.070 |  | 0.121 |
|  | GLMM-full | 0.006 | 0.026 | 0.017 | 0.045 | 0.079 |  | 0.122 |
|  | STEP | 0.004 | 0.026 | 0.017 | 0.036 | 0.073 |  | 0.071 |
|  | LASSO | 0.001 | 0.034 | 0.017 | 0.030 |  |  |  |
|  | ridge | 0.004 | 0.044 | 0.017 | 0.044 |  |  |  |
|  | adaptive LASSO | 0.002 | 0.029 | 0.017 | **0.029** |  |  |  |
|  | Bayesian LASSO | 0.002 | 0.038 | 0.017 | 0.035 | 0.080 |  | 0.184 |
|  | SSVS | 0.002 | 0.031 | 0.017 | 0.030 | 0.078 |  | 0.184 |
| C32 | GLMM-oracle | 0.000 | 0.024 | 0.076 | 0.050 | 0.071 |  | 0.258 |
|  | GLMM-full | 0.006 | 0.026 | 0.076 | 0.074 | 0.079 |  | 0.258 |
|  | STEP | 0.004 | 0.027 | 0.078 | 0.068 | 0.077 |  | 0.075 |
|  | LASSO | 0.001 | 0.036 | 0.078 | 0.062 |  |  |  |
|  | ridge | 0.004 | 0.047 | 0.078 | 0.076 |  |  |  |
|  | adaptive LASSO | 0.002 | 0.030 | 0.078 | **0.060** |  |  |  |
|  | Bayesian LASSO | 0.002 | 0.038 | 0.076 | 0.064 | 0.080 |  | 0.343 |
|  | SSVS | 0.002 | 0.031 | 0.076 | **0.060** | 0.078 |  | 0.343 |
| C33 | GLMM-oracle | 0.000 | 0.024 | 0.010 | 0.017 | 0.036 |  | 0.095 |
|  | GLMM-full | 0.002 | 0.024 | 0.010 | 0.023 | 0.040 |  | 0.095 |
|  | STEP | 0.001 | 0.024 | 0.011 | 0.021 | 0.037 |  | 0.037 |
|  | LASSO | 0.000 | 0.026 | 0.011 | 0.020 |  |  |  |
|  | ridge | 0.001 | 0.028 | 0.011 | 0.025 |  |  |  |
|  | adaptive LASSO | 0.000 | 0.025 | 0.011 | **0.019** |  |  |  |
|  | Bayesian LASSO | 0.001 | 0.026 | 0.010 | 0.021 | 0.040 |  | 0.153 |
|  | SSVS | 0.001 | 0.025 | 0.010 | 0.020 | 0.039 |  | 0.153 |
| C34 | GLMM-oracle | 0.000 | 0.024 | 0.053 | 0.039 | 0.036 |  | 0.219 |
|  | GLMM-full | 0.002 | 0.024 | 0.053 | 0.045 | 0.040 |  | 0.219 |
|  | STEP | 0.001 | 0.024 | 0.063 | 0.048 | 0.039 |  | 0.038 |
|  | LASSO | 0.000 | 0.026 | 0.063 | 0.046 |  |  |  |
|  | ridge | 0.001 | 0.029 | 0.063 | 0.051 |  |  |  |
|  | adaptive LASSO | 0.000 | 0.025 | 0.063 | 0.046 |  |  |  |
|  | Bayesian LASSO | 0.001 | 0.026 | 0.053 | 0.043 | 0.040 |  | 0.303 |
|  | SSVS | 0.001 | 0.025 | 0.053 | **0.041** | 0.039 |  | 0.303 |
| C35 | GLMM-oracle | 0.000 | 0.028 | 0.018 | 0.023 | 0.093 |  | 0.128 |
|  | GLMM-full | 0.011 | 0.031 | 0.019 | 0.159 | 0.109 |  | 0.131 |
|  | STEP | 0.007 | 0.032 | 0.019 | 0.113 | 0.096 |  | 0.093 |
|  | LASSO | 0.001 | 0.053 | 0.019 | **0.046** |  |  |  |
|  | ridge | 0.002 | 0.099 | 0.019 | 0.084 |  |  |  |
|  | adaptive LASSO | 0.002 | 0.037 | 0.019 | 0.054 |  |  |  |
|  | Bayesian LASSO | 0.001 | 0.066 | 0.019 | 0.060 | 0.103 |  | 0.192 |
|  | SSVS | 0.002 | 0.060 | 0.019 | 0.062 | 0.104 |  | 0.192 |
| C36 | GLMM-oracle | 0.000 | 0.029 | 0.062 | 0.046 | 0.093 |  | 0.231 |
|  | GLMM-full | 0.011 | 0.032 | 0.063 | 0.182 | 0.109 |  | 0.231 |
|  | STEP | 0.007 | 0.033 | 0.064 | 0.141 | 0.109 |  | 0.231 |
|  | LASSO | 0.001 | 0.055 | 0.064 | **0.070** |  |  |  |
|  | ridge | 0.002 | 0.103 | 0.064 | 0.108 |  |  |  |
|  | adaptive LASSO | 0.002 | 0.039 | 0.064 | 0.078 |  |  |  |
|  | Bayesian LASSO | 0.001 | 0.066 | 0.063 | 0.083 | 0.103 |  | 0.310 |
|  | SSVS | 0.002 | 0.060 | 0.063 | 0.085 | 0.104 |  | 0.309 |
| B1 | GLMM-oracle | 0 |  | 0.076 | 0.038 |  |  | 0.259 |
|  | GLMM-full | 0.091 |  | 0.110 | 0.459 |  |  | 0.286 |
|  | STEP | 0.052 |  | 0.096 | 0.289 |  |  | 0.268 |
|  | LASSO | 0.005 |  | 0.080 | 0.065 |  |  |  |
|  | ridge | 0.004 |  | 0.080 | **0.059** |  |  |  |
|  | adaptive LASSO | 0.014 |  | 0.082 | 0.111 |  |  |  |
|  | Bayesian LASSO | 0.002 |  | 0.106 | 0.065 |  |  | 0.395 |
|  | SSVS | 0.011 |  | 0.116 | 0.114 |  |  | 0.401 |
| B2 | GLMM-oracle | 0 |  | 0.116 | 0.058 |  |  | 0.281 |
|  | GLMM-full | 0.093 |  | 0.159 | 0.491 |  |  | 0.308 |
|  | STEP | 0.054 |  | 0.140 | 0.317 |  |  | 0.270 |
|  | LASSO | 0.006 |  | 0.128 | 0.093 |  |  |  |
|  | ridge | 0.004 |  | 0.127 | **0.084** |  |  |  |
|  | adaptive LASSO | 0.015 |  | 0.131 | 0.139 |  |  |  |
|  | Bayesian LASSO | 0.002 |  | 0.155 | 0.090 |  |  | 0.439 |
|  | SSVS | 0.012 |  | 0.165 | 0.141 |  |  | 0.444 |
| B3 | GLMM-oracle | 0 | 0.087 | 0.077 | 0.082 | 0.260 |  | 0.261 |
|  | GLMM-full | 0.088 | 0.118 | 0.106 | 0.461 | 0.297 |  | 0.285 |
|  | STEP | 0.051 | 0.082 | 0.094 | 0.297 | 0.277 |  | 0.267 |
|  | LASSO | 0.007 | 0.021 | 0.087 | 0.084 |  |  |  |
|  | ridge | 0.005 | 0.016 | 0.086 | 0.075 |  |  |  |
|  | adaptive LASSO | 0.015 | 0.033 | 0.091 | 0.128 |  |  |  |
|  | Bayesian LASSO | 0.003 | 0.011 | 0.107 | **0.072** | 0.109 |  | 0.394 |
|  | SSVS | 0.012 | 0.021 | 0.115 | 0.122 | 0.165 |  | 0.398 |
| B4 | GLMM-oracle | 0 | 0.094 | 0.125 | 0.109 | 0.262 |  | 0.282 |
|  | GLMM-full | 0.088 | 0.125 | 0.162 | 0.493 | 0.300 |  | 0.306 |
|  | STEP | 0.050 | 0.088 | 0.147 | 0.323 | 0.279 |  | 0.269 |
|  | LASSO | 0.006 | 0.020 | 0.131 | 0.102 |  |  |  |
|  | ridge | 0.005 | 0.015 | 0.130 | **0.095** |  |  |  |
|  | adaptive LASSO | 0.016 | 0.033 | 0.136 | 0.155 |  |  |  |
|  | Bayesian LASSO | 0.002 | 0.011 | 0.165 | 0.100 | 0.109 |  | 0.440 |
|  | SSVS | 0.011 | 0.026 | 0.173 | 0.150 | 0.165 |  | 0.446 |
| B5 | GLMM-oracle | 0 | 0.096 | 0.079 | 0.087 | 0.273 |  | 0.267 |
|  | GLMM-full | 0.090 | 0.124 | 0.107 | 0.476 | 0.310 |  | 0.291 |
|  | STEP | 0.054 | 0.159 | 0.098 | 0.345 | 0.287 |  | 0.272 |
|  | LASSO | 0.009 | 0.175 | 0.096 | 0.171 |  |  |  |
|  | ridge | 0.008 | 0.171 | 0.096 | 0.167 |  |  |  |
|  | adaptive LASSO | 0.019 | 0.154 | 0.099 | 0.203 |  |  |  |
|  | Bayesian LASSO | 0.005 | 0.171 | 0.123 | **0.166** | 0.164 |  | 0.401 |
|  | SSVS | 0.016 | 0.135 | 0.130 | 0.200 | 0.241 |  | 0.407 |
| B6 | GLMM-oracle | 0 | 0.095 | 0.130 | 0.112 | 0.274 |  | 0.288 |
|  | GLMM-full | 0.092 | 0.123 | 0.167 | 0.512 | 0.312 |  | 0.312 |
|  | STEP | 0.055 | 0.163 | 0.154 | 0.379 | 0.287 |  | 0.274 |
|  | LASSO | 0.009 | 0.181 | 0.144 | 0.198 |  |  |  |
|  | ridge | 0.009 | 0.174 | 0.143 | **0.194** |  |  |  |
|  | adaptive LASSO | 0.019 | 0.162 | 0.149 | 0.234 |  |  |  |
|  | Bayesian LASSO | 0.005 | 0.171 | 0.182 | 0.195 | 0.163 |  | 0.441 |
|  | SSVS | 0.016 | 0.137 | 0.189 | 0.230 | 0.240 |  | 0.448 |
| B7 | GLMM-oracle | 0 | 0.089 | 0.080 | 0.129 | 0.265 | 0.260 | 0.268 |
|  | GLMM-full | 0.105 | 0.123 | 0.143 | 0.786 | 0.312 | 0.282 | 0.306 |
|  | STEP | 0.061 | 0.083 | 0.119 | 0.507 | 0.286 | 0.277 | 0.280 |
|  | LASSO | 0.004 | 0.016 | 0.087 | 0.088 |  |  |  |
|  | ridge | 0.004 | 0.013 | 0.086 | **0.082** |  |  |  |
|  | adaptive LASSO | 0.014 | 0.029 | 0.093 | 0.164 |  |  |  |
|  | Bayesian LASSO | 0.004 | 0.013 | 0.128 | 0.101 | 0.111 | 0.109 | 0.411 |
|  | SSVS | 0.012 | 0.024 | 0.140 | 0.170 | 0.158 | 0.152 | 0.417 |
| B8 | GLMM-oracle | 0 | 0.09 | 0.128 | 0.154 | 0.267 | 0.262 | 0.291 |
|  | GLMM-full | 0.106 | 0.125 | 0.208 | 0.828 | 0.314 | 0.284 | 0.331 |
|  | STEP | 0.062 | 0.083 | 0.176 | 0.538 | 0.289 | 0.277 | 0.281 |
|  | LASSO | 0.004 | 0.016 | 0.136 | 0.109 |  |  |  |
|  | ridge | 0.003 | 0.013 | 0.134 | **0.104** |  |  |  |
|  | adaptive LASSO | 0.013 | 0.028 | 0.142 | 0.184 |  |  |  |
|  | Bayesian LASSO | 0.003 | 0.013 | 0.187 | 0.129 | 0.111 | 0.109 | 0.455 |
|  | SSVS | 0.011 | 0.023 | 0.204 | 0.199 | 0.158 | 0.153 | 0.46 |
| B9 | GLMM-oracle | 0 | 0.094 | 0.086 | 0.137 | 0.279 | 0.267 | 0.275 |
|  | GLMM-full | 0.110 | 0.130 | 0.149 | 0.825 | 0.328 | 0.291 | 0.314 |
|  | STEP | 0.067 | 0.157 | 0.134 | 0.611 | 0.298 | 0.281 | 0.288 |
|  | LASSO | 0.007 | 0.160 | 0.103 | 0.255 |  |  |  |
|  | ridge | 0.007 | 0.146 | 0.102 | **0.239** |  |  |  |
|  | adaptive LASSO | 0.017 | 0.146 | 0.105 | 0.302 |  |  |  |
|  | Bayesian LASSO | 0.007 | 0.143 | 0.165 | 0.266 | 0.169 | 0.154 | 0.419 |
|  | SSVS | 0.017 | 0.122 | 0.175 | 0.316 | 0.238 | 0.212 | 0.427 |
| B10 | GLMM-oracle | 0 | 0.096 | 0.135 | 0.164 | 0.281 | 0.269 | 0.298 |
|  | GLMM-full | 0.112 | 0.132 | 0.215 | 0.867 | 0.330 | 0.293 | 0.339 |
|  | STEP | 0.066 | 0.154 | 0.192 | 0.634 | 0.299 | 0.284 | 0.289 |
|  | LASSO | 0.007 | 0.163 | 0.145 | 0.275 |  |  |  |
|  | ridge | 0.007 | 0.150 | 0.143 | **0.262** |  |  |  |
|  | adaptive LASSO | 0.017 | 0.15 | 0.148 | 0.329 |  |  |  |
|  | Bayesian LASSO | 0.007 | 0.144 | 0.226 | 0.298 | 0.168 | 0.153 | 0.460 |
|  | SSVS | 0.017 | 0.122 | 0.239 | 0.349 | 0.237 | 0.212 | 0.469 |
| B11 | GLMM-oracle | 0 | 0.097 | 0.093 | 0.185 | 0.277 | 0.261 | 0.270 |
|  | GLMM-full | 0.103 | 0.124 | 0.173 | 0.798 | 0.311 | 0.282 | 0.305 |
|  | STEP | 0.060 | 0.084 | 0.140 | 0.514 | 0.285 | 0.272 | 0.280 |
|  | LASSO | 0.005 | 0.017 | 0.100 | 0.110 |  |  |  |
|  | ridge | 0.004 | 0.013 | 0.100 | **0.096** |  |  |  |
|  | adaptive LASSO | 0.014 | 0.029 | 0.104 | 0.183 |  |  |  |
|  | Bayesian LASSO | 0.003 | 0.012 | 0.153 | 0.115 | 0.112 | 0.109 | 0.408 |
|  | SSVS | 0.011 | 0.022 | 0.168 | 0.184 | 0.159 | 0.151 | 0.415 |
| B12 | GLMM-oracle | 0 | 0.098 | 0.135 | 0.207 | 0.279 | 0.263 | 0.287 |
|  | GLMM-full | 0.106 | 0.128 | 0.233 | 0.846 | 0.314 | 0.284 | 0.324 |
|  | STEP | 0.062 | 0.085 | 0.195 | 0.553 | 0.288 | 0.274 | 0.282 |
|  | LASSO | 0.005 | 0.016 | 0.152 | 0.132 |  |  |  |
|  | ridge | 0.004 | 0.012 | 0.151 | **0.121** |  |  |  |
|  | adaptive LASSO | 0.013 | 0.027 | 0.157 | 0.199 |  |  |  |
|  | Bayesian LASSO | 0.003 | 0.012 | 0.206 | 0.143 | 0.112 | 0.110 | 0.447 |
|  | SSVS | 0.011 | 0.022 | 0.227 | 0.217 | 0.160 | 0.154 | 0.452 |
| B13 | GLMM-oracle | 0 | 0.105 | 0.094 | 0.198 | 0.295 | 0.274 | 0.282 |
|  | GLMM-full | 0.113 | 0.139 | 0.173 | 0.871 | 0.333 | 0.298 | 0.317 |
|  | STEP | 0.067 | 0.169 | 0.155 | 0.673 | 0.308 | 0.287 | 0.293 |
|  | LASSO | 0.009 | 0.156 | 0.133 | 0.392 |  |  |  |
|  | ridge | 0.010 | 0.137 | 0.132 | **0.370** |  |  |  |
|  | adaptive LASSO | 0.018 | 0.146 | 0.132 | 0.406 |  |  |  |
|  | Bayesian LASSO | 0.009 | 0.137 | 0.231 | 0.408 | 0.194 | 0.168 | 0.424 |
|  | SSVS | 0.021 | 0.121 | 0.234 | 0.434 | 0.257 | 0.222 | 0.431 |
| B14 | GLMM-oracle | 0 | 0.107 | 0.137 | 0.224 | 0.297 | 0.275 | 0.301 |
|  | GLMM-full | 0.114 | 0.142 | 0.228 | 0.912 | 0.335 | 0.299 | 0.338 |
|  | STEP | 0.069 | 0.171 | 0.204 | 0.711 | 0.309 | 0.288 | 0.294 |
|  | LASSO | 0.008 | 0.159 | 0.186 | 0.419 |  |  |  |
|  | ridge | 0.009 | 0.14 | 0.184 | **0.398** |  |  |  |
|  | adaptive LASSO | 0.017 | 0.147 | 0.187 | 0.431 |  |  |  |
|  | Bayesian LASSO | 0.009 | 0.141 | 0.278 | 0.438 | 0.190 | 0.167 | 0.457 |
|  | SSVS | 0.020 | 0.124 | 0.286 | 0.464 | 0.255 | 0.221 | 0.466 |
| B15 | GLMM-oracle | 0 |  | 0.037 | 0.018 |  |  | 0.178 |
|  | GLMM-full | 0.040 |  | 0.045 | 0.202 |  |  | 0.188 |
|  | STEP | 0.023 |  | 0.042 | 0.129 |  |  | 0.181 |
|  | LASSO | 0.003 |  | 0.037 | 0.031 |  |  |  |
|  | ridge | 0.002 |  | 0.037 | **0.028** |  |  |  |
|  | adaptive LASSO | 0.007 |  | 0.038 | 0.053 |  |  |  |
|  | Bayesian LASSO | 0.002 |  | 0.045 | 0.031 |  |  | 0.236 |
|  | SSVS | 0.005 |  | 0.046 | 0.047 |  |  | 0.237 |
| B16 | GLMM-oracle | 0 |  | 0.061 | 0.031 |  |  | 0.190 |
|  | GLMM-full | 0.040 |  | 0.072 | 0.216 |  |  | 0.200 |
|  | STEP | 0.023 |  | 0.069 | 0.142 |  |  | 0.183 |
|  | LASSO | 0.003 |  | 0.059 | 0.044 |  |  |  |
|  | ridge | 0.002 |  | 0.059 | **0.040** |  |  |  |
|  | adaptive LASSO | 0.007 |  | 0.061 | 0.066 |  |  |  |
|  | Bayesian LASSO | 0.002 |  | 0.074 | 0.046 |  |  | 0.276 |
|  | SSVS | 0.005 |  | 0.076 | 0.062 |  |  | 0.277 |
| B17 | GLMM-oracle | 0 | 0.044 | 0.034 | 0.039 | 0.180 |  | 0.180 |
|  | GLMM-full | 0.038 | 0.054 | 0.041 | 0.197 | 0.200 |  | 0.188 |
|  | STEP | 0.022 | 0.042 | 0.038 | 0.130 | 0.187 |  | 0.182 |
|  | LASSO | 0.003 | 0.012 | 0.037 | 0.037 |  |  |  |
|  | ridge | 0.002 | 0.010 | 0.037 | **0.034** |  |  |  |
|  | adaptive LASSO | 0.007 | 0.018 | 0.038 | 0.059 |  |  |  |
|  | Bayesian LASSO | 0.002 | 0.010 | 0.045 | **0.034** | 0.086 |  | 0.239 |
|  | SSVS | 0.004 | 0.014 | 0.046 | 0.049 | 0.111 |  | 0.241 |
| B18 | GLMM-oracle | 0 | 0.044 | 0.057 | 0.051 | 0.181 |  | 0.193 |
|  | GLMM-full | 0.038 | 0.056 | 0.067 | 0.213 | 0.202 |  | 0.202 |
|  | STEP | 0.021 | 0.042 | 0.063 | 0.140 | 0.187 |  | 0.183 |
|  | LASSO | 0.003 | 0.013 | 0.060 | 0.049 |  |  |  |
|  | ridge | 0.002 | 0.010 | 0.060 | **0.045** |  |  |  |
|  | adaptive LASSO | 0.007 | 0.018 | 0.061 | 0.072 |  |  |  |
|  | Bayesian LASSO | 0.002 | 0.010 | 0.072 | 0.048 | 0.086 |  | 0.277 |
|  | SSVS | 0.004 | 0.014 | 0.074 | 0.063 | 0.112 |  | 0.278 |
| B19 | GLMM-oracle | 0 | 0.050 | 0.037 | 0.044 | 0.189 |  | 0.183 |
|  | GLMM-full | 0.039 | 0.060 | 0.044 | 0.207 | 0.209 |  | 0.191 |
|  | STEP | 0.023 | 0.078 | 0.042 | 0.151 | 0.195 |  | 0.185 |
|  | LASSO | 0.004 | 0.127 | 0.042 | **0.102** |  |  |  |
|  | ridge | 0.006 | 0.143 | 0.043 | 0.114 |  |  |  |
|  | adaptive LASSO | 0.009 | 0.095 | 0.042 | 0.104 |  |  |  |
|  | Bayesian LASSO | 0.004 | 0.132 | 0.057 | 0.109 | 0.156 |  | 0.242 |
|  | SSVS | 0.007 | 0.101 | 0.055 | 0.108 | 0.192 |  | 0.244 |
| B20 | GLMM-oracle | 0 | 0.051 | 0.057 | 0.054 | 0.190 |  | 0.195 |
|  | GLMM-full | 0.040 | 0.060 | 0.067 | 0.222 | 0.210 |  | 0.203 |
|  | STEP | 0.023 | 0.082 | 0.064 | 0.165 | 0.195 |  | 0.186 |
|  | LASSO | 0.005 | 0.133 | 0.063 | **0.117** |  |  |  |
|  | ridge | 0.005 | 0.150 | 0.063 | 0.127 |  |  |  |
|  | adaptive LASSO | 0.009 | 0.102 | 0.063 | 0.119 |  |  |  |
|  | Bayesian LASSO | 0.004 | 0.134 | 0.081 | 0.122 | 0.155 |  | 0.278 |
|  | SSVS | 0.007 | 0.102 | 0.080 | 0.121 | 0.191 |  | 0.281 |
| B21 | GLMM-oracle | 0 | 0.042 | 0.039 | 0.062 | 0.181 | 0.179 | 0.182 |
|  | GLMM-full | 0.041 | 0.051 | 0.052 | 0.311 | 0.204 | 0.185 | 0.194 |
|  | STEP | 0.024 | 0.038 | 0.048 | 0.203 | 0.191 | 0.183 | 0.185 |
|  | LASSO | 0.002 | 0.011 | 0.038 | 0.045 |  |  |  |
|  | ridge | 0.002 | 0.010 | 0.038 | **0.040** |  |  |  |
|  | adaptive LASSO | 0.006 | 0.016 | 0.040 | 0.077 |  |  |  |
|  | Bayesian LASSO | 0.002 | 0.010 | 0.055 | 0.049 | 0.083 | 0.083 | 0.242 |
|  | SSVS | 0.004 | 0.012 | 0.055 | 0.065 | 0.099 | 0.099 | 0.244 |
| B22 | GLMM-oracle | 0 | 0.043 | 0.062 | 0.074 | 0.183 | 0.180 | 0.193 |
|  | GLMM-full | 0.042 | 0.052 | 0.079 | 0.333 | 0.206 | 0.186 | 0.205 |
|  | STEP | 0.024 | 0.038 | 0.074 | 0.220 | 0.191 | 0.183 | 0.186 |
|  | LASSO | 0.002 | 0.011 | 0.060 | 0.057 |  |  |  |
|  | ridge | 0.002 | 0.010 | 0.060 | **0.052** |  |  |  |
|  | adaptive LASSO | 0.007 | 0.016 | 0.062 | 0.090 |  |  |  |
|  | Bayesian LASSO | 0.002 | 0.010 | 0.083 | 0.063 | 0.083 | 0.084 | 0.278 |
|  | SSVS | 0.004 | 0.012 | 0.085 | 0.080 | 0.100 | 0.100 | 0.281 |
| B23 | GLMM-oracle | 0 | 0.046 | 0.041 | 0.067 | 0.191 | 0.183 | 0.186 |
|  | GLMM-full | 0.043 | 0.054 | 0.053 | 0.325 | 0.214 | 0.191 | 0.198 |
|  | STEP | 0.025 | 0.077 | 0.050 | 0.246 | 0.199 | 0.187 | 0.190 |
|  | LASSO | 0.004 | 0.118 | 0.049 | **0.168** |  |  |  |
|  | ridge | 0.005 | 0.120 | 0.049 | 0.174 |  |  |  |
|  | adaptive LASSO | 0.008 | 0.098 | 0.048 | 0.169 |  |  |  |
|  | Bayesian LASSO | 0.004 | 0.115 | 0.073 | 0.175 | 0.148 | 0.136 | 0.249 |
|  | SSVS | 0.007 | 0.094 | 0.071 | 0.171 | 0.181 | 0.165 | 0.251 |
| B24 | GLMM-oracle | 0 | 0.047 | 0.065 | 0.079 | 0.192 | 0.184 | 0.197 |
|  | GLMM-full | 0.043 | 0.054 | 0.082 | 0.343 | 0.215 | 0.192 | 0.209 |
|  | STEP | 0.025 | 0.080 | 0.078 | 0.267 | 0.199 | 0.188 | 0.191 |
|  | LASSO | 0.004 | 0.121 | 0.069 | **0.180** |  |  |  |
|  | ridge | 0.005 | 0.124 | 0.069 | 0.187 |  |  |  |
|  | adaptive LASSO | 0.008 | 0.098 | 0.068 | 0.183 |  |  |  |
|  | Bayesian LASSO | 0.004 | 0.118 | 0.104 | 0.193 | 0.146 | 0.135 | 0.282 |
|  | SSVS | 0.007 | 0.096 | 0.103 | 0.189 | 0.180 | 0.164 | 0.284 |
| B25 | GLMM-oracle | 0 | 0.045 | 0.041 | 0.084 | 0.190 | 0.179 | 0.183 |
|  | GLMM-full | 0.041 | 0.050 | 0.052 | 0.314 | 0.204 | 0.185 | 0.194 |
|  | STEP | 0.023 | 0.038 | 0.048 | 0.207 | 0.190 | 0.182 | 0.185 |
|  | LASSO | 0.003 | 0.012 | 0.044 | 0.057 |  |  |  |
|  | ridge | 0.002 | 0.010 | 0.043 | **0.052** |  |  |  |
|  | adaptive LASSO | 0.007 | 0.018 | 0.044 | 0.089 |  |  |  |
|  | Bayesian LASSO | 0.002 | 0.009 | 0.056 | 0.054 | 0.084 | 0.084 | 0.241 |
|  | SSVS | 0.003 | 0.012 | 0.058 | 0.070 | 0.102 | 0.101 | 0.244 |
| B26 | GLMM-oracle | 0 | 0.046 | 0.060 | 0.095 | 0.191 | 0.180 | 0.195 |
|  | GLMM-full | 0.041 | 0.051 | 0.075 | 0.330 | 0.205 | 0.186 | 0.205 |
|  | STEP | 0.023 | 0.038 | 0.070 | 0.219 | 0.190 | 0.183 | 0.186 |
|  | LASSO | 0.002 | 0.012 | 0.065 | 0.067 |  |  |  |
|  | ridge | 0.002 | 0.010 | 0.065 | **0.062** |  |  |  |
|  | adaptive LASSO | 0.007 | 0.017 | 0.066 | 0.100 |  |  |  |
|  | Bayesian LASSO | 0.002 | 0.010 | 0.080 | 0.066 | 0.084 | 0.084 | 0.277 |
|  | SSVS | 0.004 | 0.012 | 0.082 | 0.082 | 0.103 | 0.101 | 0.278 |
| B27 | GLMM-oracle | 0 | 0.051 | 0.043 | 0.097 | 0.202 | 0.187 | 0.191 |
|  | GLMM-full | 0.043 | 0.056 | 0.053 | 0.340 | 0.217 | 0.194 | 0.202 |
|  | STEP | 0.025 | 0.084 | 0.053 | 0.282 | 0.205 | 0.191 | 0.194 |
|  | LASSO | 0.005 | 0.109 | 0.058 | 0.242 |  |  |  |
|  | ridge | 0.008 | 0.102 | 0.06 | 0.253 |  |  |  |
|  | adaptive LASSO | 0.009 | 0.093 | 0.054 | **0.223** |  |  |  |
|  | Bayesian LASSO | 0.007 | 0.101 | 0.085 | 0.255 | 0.168 | 0.148 | 0.254 |
|  | SSVS | 0.009 | 0.086 | 0.081 | 0.232 | 0.196 | 0.173 | 0.255 |
| B28 | GLMM-oracle | 0 | 0.052 | 0.064 | 0.109 | 0.202 | 0.188 | 0.203 |
|  | GLMM-full | 0.044 | 0.057 | 0.078 | 0.357 | 0.218 | 0.195 | 0.214 |
|  | STEP | 0.025 | 0.084 | 0.076 | 0.296 | 0.205 | 0.191 | 0.195 |
|  | LASSO | 0.005 | 0.113 | 0.081 | 0.264 |  |  |  |
|  | ridge | 0.008 | 0.107 | 0.082 | 0.274 |  |  |  |
|  | adaptive LASSO | 0.009 | 0.099 | 0.078 | **0.246** |  |  |  |
|  | Bayesian LASSO | 0.006 | 0.105 | 0.112 | 0.273 | 0.166 | 0.147 | 0.284 |
|  | SSVS | 0.009 | 0.088 | 0.107 | 0.248 | 0.196 | 0.172 | 0.287 |
| B29 | GLMM-oracle |  | 0.137 | 0.130 | 0.715 | 0.335 | 0.305 | 0.325 |
|  | GLMM-full |  | 0.137 | 0.130 | **0.715** | 0.335 | 0.305 | 0.325 |
|  | STEP |  | 0.172 | 0.152 | 0.900 | 0.319 | 0.298 | 0.301 |
|  | LASSO |  | 0.141 | 0.178 | 0.930 |  |  |  |
|  | ridge |  | 0.110 | 0.157 | 0.753 |  |  |  |
|  | adaptive LASSO |  | 0.148 | 0.170 | 0.914 |  |  |  |
|  | Bayesian LASSO |  | 0.127 | 0.281 | 0.923 | 0.227 | 0.202 | 0.440 |
|  | SSVS |  | 0.124 | 0.257 | 0.811 | 0.277 | 0.240 | 0.443 |
| B30 | GLMM-oracle |  | 0.138 | 0.179 | 0.746 | 0.336 | 0.306 | 0.342 |
|  | GLMM-full |  | 0.138 | 0.179 | **0.746** | 0.336 | 0.306 | 0.342 |
|  | STEP |  | 0.173 | 0.198 | 0.936 | 0.319 | 0.298 | 0.302 |
|  | LASSO |  | 0.145 | 0.219 | 0.984 |  |  |  |
|  | ridge |  | 0.113 | 0.198 | 0.802 |  |  |  |
|  | adaptive LASSO |  | 0.150 | 0.214 | 0.960 |  |  |  |
|  | Bayesian LASSO |  | 0.129 | 0.324 | 0.964 | 0.223 | 0.199 | 0.462 |
|  | SSVS |  | 0.125 | 0.302 | 0.846 | 0.275 | 0.238 | 0.470 |
| B31 | GLMM-oracle | 0.000 | 0.091 | 0.083 | 0.086 | 0.261 |  | 0.263 |
|  | GLMM-full | 0.087 | 0.122 | 0.116 | 0.464 | 0.299 |  | 0.288 |
|  | STEP | 0.050 | 0.083 | 0.104 | 0.300 | 0.276 |  | 0.268 |
|  | LASSO | 0.006 | 0.018 | 0.088 | 0.079 |  |  |  |
|  | ridge | 0.005 | 0.014 | 0.088 | **0.072** |  |  |  |
|  | adaptive LASSO | 0.016 | 0.031 | 0.092 | 0.130 |  |  |  |
|  | Bayesian LASSO | 0.003 | 0.011 | 0.115 | 0.076 | 0.109 |  | 0.402 |
|  | SSVS | 0.012 | 0.024 | 0.125 | 0.130 | 0.166 |  | 0.407 |
| B32 | GLMM-oracle | 0.000 | 0.093 | 0.156 | 0.124 | 0.264 |  | 0.294 |
|  | GLMM-full | 0.090 | 0.128 | 0.203 | 0.521 | 0.302 |  | 0.320 |
|  | STEP | 0.051 | 0.086 | 0.182 | 0.344 | 0.277 |  | 0.271 |
|  | LASSO | 0.006 | 0.019 | 0.162 | 0.117 |  |  |  |
|  | Ridge | 0.005 | 0.014 | 0.160 | **0.109** |  |  |  |
|  | adaptive LASSO | 0.016 | 0.031 | 0.166 | 0.167 |  |  |  |
|  | Bayesian LASSO | 0.003 | 0.011 | 0.199 | 0.118 | 0.110 |  | 0.460 |
|  | SSVS | 0.012 | 0.025 | 0.210 | 0.172 | 0.167 |  | 0.464 |
| B33 | GLMM-oracle | 0.000 | 0.040 | 0.024 | 0.032 | 0.134 |  | 0.140 |
|  | GLMM-full | 0.021 | 0.043 | 0.027 | 0.119 | 0.147 |  | 0.145 |
|  | STEP | 0.012 | 0.036 | 0.026 | 0.082 | 0.136 |  | 0.134 |
|  | LASSO | 0.002 | 0.016 | 0.025 | 0.029 |  |  |  |
|  | ridge | 0.002 | 0.011 | 0.025 | **0.026** |  |  |  |
|  | adaptive LASSO | 0.005 | 0.022 | 0.025 | 0.044 |  |  |  |
|  | Bayesian LASSO | 0.001 | 0.011 | 0.028 | **0.026** | 0.076 |  | 0.250 |
|  | SSVS | 0.002 | 0.015 | 0.029 | 0.033 | 0.090 |  | 0.250 |
| B34 | GLMM-oracle | 0.000 | 0.039 | 0.067 | 0.053 | 0.135 |  | 0.200 |
|  | GLMM-full | 0.021 | 0.043 | 0.072 | 0.143 | 0.149 |  | 0.204 |
|  | STEP | 0.012 | 0.036 | 0.073 | 0.106 | 0.136 |  | 0.135 |
|  | LASSO | 0.002 | 0.015 | 0.071 | 0.053 |  |  |  |
|  | ridge | 0.002 | 0.011 | 0.071 | 0.049 |  |  |  |
|  | adaptive LASSO | 0.005 | 0.022 | 0.072 | 0.069 |  |  |  |
|  | Bayesian LASSO | 0.001 | 0.011 | 0.072 | **0.048** | 0.077 |  | 0.338 |
|  | SSVS | 0.003 | 0.015 | 0.072 | 0.055 | 0.091 |  | 0.339 |
| B35 | GLMM-oracle | 0.000 | 0.110 | 0.094 | 0.102 | 0.283 |  | 0.280 |
|  | GLMM-full | 0.173 | 0.248 | 0.401 | 2.465 | 0.381 |  | 0.394 |
|  | STEP | 0.098 | 0.235 | 0.258 | 1.501 | 0.330 |  | 0.323 |
|  | LASSO | 0.003 | 0.212 | 0.124 | 0.208 |  |  |  |
|  | ridge | 0.002 | 0.208 | 0.123 | **0.199** |  |  |  |
|  | adaptive LASSO | 0.012 | 0.185 | 0.133 | 0.324 |  |  |  |
|  | Bayesian LASSO | 0.008 | 0.178 | 0.270 | 0.341 | 0.176 |  | 0.465 |
|  | SSVS | 0.019 | 0.155 | 0.307 | 0.508 | 0.235 |  | 0.477 |
| B36 | GLMM-oracle | 0.000 | 0.107 | 0.136 | 0.121 | 0.284 |  | 0.303 |
|  | GLMM-full | 0.179 | 0.250 | 0.491 | 2.584 | 0.394 |  | 0.424 |
|  | STEP | 0.099 | 0.227 | 0.317 | 1.543 | 0.384 |  | 0.325 |
|  | LASSO | 0.003 | 0.213 | 0.168 | 0.233 |  |  |  |
|  | ridge | 0.002 | 0.209 | 0.168 | **0.222** |  |  |  |
|  | adaptive LASSO | 0.012 | 0.186 | 0.179 | 0.352 |  |  |  |
|  | Bayesian LASSO | 0.008 | 0.177 | 0.334 | 0.373 | 0.176 |  | 0.506 |
|  | SSVS | 0.019 | 0.154 | 0.379 | 0.547 | 0.235 |  | 0.519 |

# Example of R code used for fitting the various meta-analysis models

## ***Generate example datasets***

Here we generate two mock datasets. The first has a continuous outcome (y) and two continuous covariates (z1, z2). The second dataset has a binary outcome (x), one continuous covariate (w1) and one binary (w2).

# The github library ("bipd") contains functions for generating sample data and running Bayesian IPD-MA methods.

library(devtools)

devtools::install_github("MikeJSeo/bipd") #parallel packages take a while to install

library(bipd)

##load data

ds <- generate_ipdma_example(type = "continuous")

ds2 <- generate_ipdma_example(type = "binary")

head(ds)

head(ds2)

## ***Code for fitting GLMM***

# continuous outcome

library(lme4) #for fitting glmm

m1 <- lmer(y ~ studyid + (z1+z2)*treat + (-1 + treat|studyid), data = ds)

summary(m1)

# estimating treatment effect for specific values of the covariates

contr <- c(rep(0, 8), 1, 1, 0.5) #if covariates are standardized this needs to be modified

v1 <- vcov(m1)

se1 <- c(sqrt(contr %*% v1 %*% contr))

mean1 <- c(contr %*% summary(m1)$coefficients[,"Estimate"])

mean1 + qnorm(c(.025,0.5,.975))* as.vector(se1[[1]])

# binary outcome

m2 <- glmer(y ~ studyid + (w1+w2)*treat + (-1 + treat|studyid), data = ds2, family = binomial)

summary(m2)

# estimating treatment effect for specific values of the covariates

contr <- c(rep(0, 8), 1, 1, 0.5) #if covariates are standardized this needs to be modified

v1 <- vcov(m2)

se1 <- c(sqrt(contr %*% v1 %*% contr))

mean1 <- c(contr %*% summary(m2)$coefficients[,"Estimate"])

exp(mean1 + qnorm(c(.025,0.5,.975))* as.vector(se1[[1]])) #calculate odds ratio

## ***Code for fitting STEP***

# continuous outcome

m3 <- glm(y ~ studyid + (z1+z2)*treat, data = ds) #glm model without mixed effects

s1 <- step(m3, scope=list(lower = ~ z1+z2+treat), direction = "both")

summary(s1)

# binary outcome

m4 <- glm(y ~ studyid + (w1+w2)*treat, family = binomial(link = "logit"), data = ds2)

s2 <- step(m4, scope=list(lower = ~ w1+w2+treat), direction = "both")

summary(s2)

## ***Code for fitting LASSO***

# continuous outcome

library(glmnet)

p.fac <- c(rep(0, 5), rep(0, 2), 0, rep(1,2)) # Shrinkage is only on effect modifiers

lambdas <- 10^seq(3, -3, by = -.1) # manually specify lambda value to cross validate

data_glmnet <- model.matrix(y~ studyid + (z1+z2)*treat, data = ds)

data_glmnet <- data_glmnet[,-1]

data_glmnet <- cbind(y = ds$y, data_glmnet = data_glmnet)

cvfit <- cv.glmnet(as.matrix(data_glmnet[,-1]), as.matrix(data_glmnet[,1]), penalty.factor = p.fac, family = "gaussian", type.measure = "deviance", lambda = lambdas)

coef(cvfit, s = "lambda.min")

# binary outcome

data_glmnet <- model.matrix(y~ studyid + (w1+w2)*treat, data = ds2)

data_glmnet <- data_glmnet[,-1]

data_glmnet <- cbind(y = ds2$y, data_glmnet = data_glmnet)

cvfit <- cv.glmnet(as.matrix(data_glmnet[,-1]), as.matrix(data_glmnet[,1]), penalty.factor = p.fac, family = "binomial", type.measure = "deviance", lambda = lambdas)

coef(cvfit, s = "lambda.min")

## ***Code for fitting ridge***

# continuous outcome

data_glmnet <- model.matrix(y~ studyid + (z1+z2)*treat, data = ds)

data_glmnet <- data_glmnet[,-1]

data_glmnet <- cbind(y = ds$y, data_glmnet = data_glmnet)

cvfit.ridge = cv.glmnet(as.matrix(data_glmnet[,-1]), as.matrix(data_glmnet[,1]), penalty.factor = p.fac, family = "gaussian", alpha = 0, type.measure = "deviance", lambda = lambdas)

coef(cvfit.ridge, s = "lambda.min")

# binary outcome

data_glmnet <- model.matrix(y~ studyid + (w1+w2)*treat, data = ds2)

data_glmnet <- data_glmnet[,-1]

data_glmnet <- cbind(y = ds2$y, data_glmnet = data_glmnet)

cvfit.ridge2 = cv.glmnet(as.matrix(data_glmnet[,-1]), as.matrix(data_glmnet[,1]), penalty.factor = p.fac, family = "binomial", alpha = 0, type.measure = "deviance", lambda = lambdas)

coef(cvfit.ridge2, s = "lambda.min")

## ***Code for fitting adaptive LASSO***

# continuous outcome

ridge_result <- coef(cvfit.ridge, s = "lambda.min")[-1]

p.fac2 <- p.fac/ abs(ridge_result)

data_glmnet <- model.matrix(y~ studyid + (z1+z2)*treat, data = ds)

data_glmnet <- data_glmnet[,-1]

data_glmnet <- cbind(y = ds$y, data_glmnet = data_glmnet)

cvfit <- cv.glmnet(as.matrix(data_glmnet[,-1]), as.matrix(data_glmnet[,1]), penalty.factor = p.fac2, family = "gaussian", type.measure = "deviance", lambda = lambdas)

coef(cvfit, s = "lambda.min")

# binary outcome

ridge_result <- coef(cvfit.ridge2, s = "lambda.min")[-1]

p.fac2 <- p.fac/ abs(ridge_result)

data_glmnet <- model.matrix(y~ studyid + (w1+w2)*treat, data = ds2)

data_glmnet <- data_glmnet[,-1]

data_glmnet <- cbind(y = ds2$y, data_glmnet = data_glmnet)

cvfit <- cv.glmnet(as.matrix(data_glmnet[,-1]), as.matrix(data_glmnet[,1]), penalty.factor = p.fac2, family = "binomial", type.measure = "deviance", lambda = lambdas)

coef(cvfit, s = "lambda.min")

## ***Code for fitting Bayesian LASSO***

# Stored variable names are as follows:

#"beta" - coefficients for main effects of the covariates

#"gamma" - coefficients for effect modifiers

#"delta" - average treaetment effect

#"lambda" - shrinkage parameter

# continuous outcome

ipd <- with(ds, ipdma.model.onestage(y = y, study = studyid, treat = treat, X = cbind(z1, z2), response = "normal", shrinkage = "laplace", lambda.prior = list("dgamma",2,0.1)))

##To see the JAGS code used to run the model use the command:

cat(ipd$code)

samples <- ipd.run(ipd, pars.save = c("lambda", "beta", "gamma", "delta"), n.chains = 3, n.burnin = 500, n.iter = 5000)

samples <- samples[,-3] #remove delta[1] which is 0

summary(samples)

plot(samples) #traceplot and posterior of parameters

coda::gelman.plot(samples) #gelman diagnostic plot

# can also find treatment effect

treatment.effect(ipd, samples, newpatient = c(1,0.5))

# binary outcome

ipd <- with(ds2, ipdma.model.onestage(y = y, study = studyid, treat = treat, X = cbind(w1, w2), response = "binomial", shrinkage = "laplace"))

samples <- ipd.run(ipd, pars.save = c("lambda", "beta", "gamma", "delta"))

summary(samples)

# can also run methods in parallel using dclone package

samples2 <- ipd.run.parallel(ipd, pars.save = c("lambda", "beta", "gamma", "delta"))

summary(samples2)

## ***Code for fitting SSVS***

# Stored variable names are as follows:

#"beta" - coefficients for main effects of the covariates

#"gamma" - coefficients for effect modifiers

#"delta" - coefficient of average treatment effect

#"Ind" - Indicator for assigning a slab prior (instead of a spike prior) i.e. indicator for including a covariate

#"eta" - Standard deviation of the slab prior

# continuous outcome

ipd <- with(ds, ipdma.model.onestage(y = y, study = studyid, treat = treat, X = cbind(z1, z2), response = "normal", shrinkage = "SSVS", hy.prior.eta = list("dunif", 0, 5), g = 1000))

samples <- ipd.run(ipd, pars.save = c("beta", "gamma", "delta", "Ind", "eta"))

samples <- samples[,-5]

summary(samples)

plot(samples)

coda::gelman.plot(samples)

# binary outcome

ipd <- with(ds2, ipdma.model.onestage(y = y, study = studyid, treat = treat, X = cbind(w1, w2), response = "binomial", shrinkage = "SSVS"))

samples <- ipd.run(ipd, pars.save = c("beta", "gamma", "delta", "Ind", "eta"))

summary(samples)

treatment.effect(ipd, samples, newpatient = c(1,0.5)) # binary outcome reports odds ratio
